# Toward a new generation of agricultural system data, models, and knowledge products: State of agricultural systems science

**DOI:** 10.1016/j.agsy.2016.09.021

**Published:** 2017-07

**Authors:** James W. Jones, John M. Antle, Bruno Basso, Kenneth J. Boote, Richard T. Conant, Ian Foster, H. Charles J. Godfray, Mario Herrero, Richard E. Howitt, Sander Janssen, Brian A. Keating, Rafael Munoz-Carpena, Cheryl H. Porter, Cynthia Rosenzweig, Tim R. Wheeler

**Affiliations:** a University of Florida, Agricultural and Biological Engineering Department, Museum Road, Gainesville, FL 32611, USA; b Oregon State University, USA; c Michigan State University, USA; d Colorado State University, USA; e University of Chicago and Argonne National Laboratory, USA; f Oxford Martin Programme on the Future of Food, University of Oxford, Department of Zoology, South Parks Rd., Oxford OX1 3PS, UK; g CSIRO, Australia; h University of California-Davis, USA; i Wageningen University, Netherlands; j NASA/Columbia University, USA; k University of Reading, UK

**Keywords:** Integrated agricultural systems models, Crop models, Economic models, Livestock models, Use cases, Agricultural data

## Abstract

We review the current state of agricultural systems science, focusing in particular on the capabilities and limitations of agricultural systems models. We discuss the state of models relative to five different Use Cases spanning field, farm, landscape, regional, and global spatial scales and engaging questions in past, current, and future time periods. Contributions from multiple disciplines have made major advances relevant to a wide range of agricultural system model applications at various spatial and temporal scales. Although current agricultural systems models have features that are needed for the Use Cases, we found that all of them have limitations and need to be improved. We identified common limitations across all Use Cases, namely 1) a scarcity of data for developing, evaluating, and applying agricultural system models and 2) inadequate knowledge systems that effectively communicate model results to society. We argue that these limitations are greater obstacles to progress than gaps in conceptual theory or available methods for using system models. New initiatives on open data show promise for addressing the data problem, but there also needs to be a cultural change among agricultural researchers to ensure that data for addressing the range of Use Cases are available for future model improvements and applications. We conclude that multiple platforms and multiple models are needed for model applications for different purposes. The Use Cases provide a useful framework for considering capabilities and limitations of existing models and data.

## Highlights

• Data limitations exist for all components of agricultural systems.
• Model limitations also exist, more severely in constrained, complex systems.
• Knowledge products for informing decisions and policy remain very limited.
• Use cases provide an important context for model evaluation and improvement.
• More emphasis is needed on models, systems analyses of agricultural systems.

## 1 Introduction

Agricultural systems science as we know it today has evolved over the last 50 or more years with contributions from a wide range of disciplines (Jones et al., this issue). Generally during this same time period, appreciation for and acceptance of agricultural systems science has increased as more scientists, engineers, and economists graduate from universities with training in systems modeling, analytical approaches, and information technology (IT) tools. Over this time period, there has also been a corresponding increase in demands for agricultural systems science to address questions faced by society that transcend agriculture. Relevant questions range from how to better manage systems for higher and more efficient production, what changes are needed in a farming system for higher profitability without harming the environment, what policies are needed to help farming systems evolve to meet broader societal goals, and what systems are needed to adapt to the continual changes that agriculture faces, including climate change, changes in demand for agricultural products, volatile energy prices, and limitations of land, water, and other natural resources. Agricultural systems models are being challenged to move beyond just including economic and sustainability issues. There is a strong agenda of new Sustainable Development Goals (e.g., FAO, 2016), which will require models of nutritional quality of food beyond bulk yields and multifunctional landscape models for policy analyses. Sustainable solutions that address multiple goals will likely benefit from a convergence of science and technologies that make use of information and cognitive sciences (Scott et al., 2015, Wolfe et al., 2016).

In order to analyze these different dimensions of agriculture and food systems, ideally we would have a virtual laboratory containing models, data, analytical tools and IT tools to conduct studies that evaluate outcomes and tradeoffs among alternative technologies, policies, or scenarios. The virtual laboratory would allow users to define scenarios, specify analyses covering different social, political, and resource situations and different spatial and temporal scales, and produce outputs suitable for interpretation and use by decision makers. Clearly, that virtual laboratory does not exist. But where are we currently relative to this ideal situation? The purpose of this paper is to address that question by reviewing the state of agricultural systems science and its capabilities for the Use Cases described by Antle, Jones and Rosenzweig (this issue) that represent two important areas of agricultural systems model applications: for smallholder agriculture in developing countries and for commercial agriculture in industrialized countries. This paper builds on earlier reviews of specific components. In the concluding article of this Special Issue, Antle, Jones and Rosenzweig (this issue) discuss the implications of NextGen for global change research, another major area of agricultural systems model applications.

## 2 Component agricultural system models

Here, we address models as components of integrated agricultural systems models, focusing on applicability of models for selected Use Cases. Janssen et al. (this issue) discuss the capabilities and limitations of various data and information tools for the different Use Cases as well as what is needed for the next generation of models and knowledge systems.

### 2.1 Cropping system and grassland models

Several crop modeling review papers have recently been published (e.g., Holzworth et al., 2015, Boote et al., 2013, Basso et al., 2016), summarizing model capabilities and uses. For example, Rivington and Koo (2010) surveyed crop model developers and users to assess the state of crop models for use in research and decision making related to climate change. They emphasized the need for additional model development as well as the need for more and better quality data. Ewert et al. (2014) reviewed crop models relative to their adequacy in performing integrated assessments of climate change impacts, and pointed out important limitations in most crop models. Holzworth et al. (2015) discussed advances in capabilities and applications over time. Basso et al. (2016) reviewed the performance of CERES maize (Ritchie, 1986), wheat (Otter and Ritchie, 1985) and rice models (Ritchie et al., 1986a) compared to measured data over the last 30 years in 43 countries. They reported that model performance, using site-specific inputs, was outstanding for the variables compared (e.g., average relative error for grain yield of 13%).

Models of cropping and grassland systems share the same fundament characteristics: both describe crop or grassland agro-ecosystem growth and yield responses to climate, soil, plant species characteristics, and management. However, several aspects of grassland/rangeland modeling present unique challenges. Many of these challenges stem from the requirement that grassland models represent several interacting species, including perennial and woody species of grasses. Persistence of plants over multiple years forces the models to consider residual effects over time. Dependency on soil-derived nutrients or human-induced disturbances like fire reinforce the longer-term perspective needed for grassland modeling. Thus, although most biophysical processes are similar (e.g., relative to photosynthesis, growth, water and nutrient uptake from soil, etc.) additional factors are considered when modeling grasslands.

#### 2.1.1 Model-simulated responses of interest to users

The most common response variable modeled for cropping systems is yield, whether of grain, tuber, or forage biomass yield. This yield is harvested at a single point in time for determinate annual crops, while indeterminate crops and grasslands may be harvested multiple times. Although statistical models may be useful for predicting these biological yields in response to some combination of weather conditions, nutrient levels, irrigation amounts, etc. (e.g., Schlenker and Lobell, 2010, Lobell et al., 2011), they do not predict responses to nonlinearities and threshold effects outside the range of conditions in data used to develop them.

In contrast, dynamic cropping and grassland system models may simulate these biological yields and other responses important to analysts, such as crop water use, nitrogen uptake, nitrate leaching, soil erosion, soil carbon, greenhouse gas emissions, and residual soil nutrients. Dynamic models can also be used to estimate responses in places and for time periods and conditions for which there are no prior experiments. They can be used to simulate experiments and estimate responses that allow users to evaluate economic and environmental tradeoffs among alternative systems. Simulation experiments can predict responses to various climate and soil conditions, genetics, and management factors that are represented in the model. “Hybrid” agricultural system models that combine dynamic crop simulations with appropriate economic models can simulate policy-relevant “treatment effects” in an experimental design of climate impact and adaptation (Antle and Stockle, 2015).

#### 2.1.2 Factors to which cropping and grassland systems respond

**Fig. 1:**
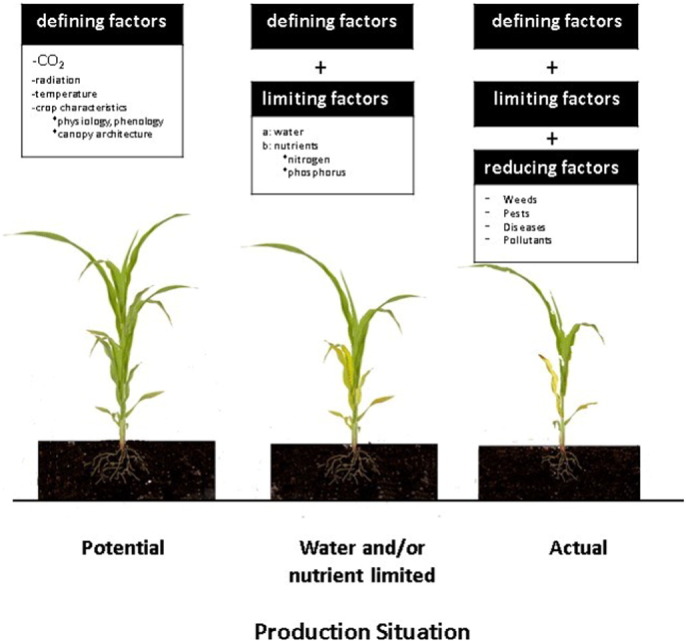
Diagram of production situation used to characterize factors included and excluded from cropping system models to help guide their development and inform users of their applicability to address different questions.

Adapted from van Ittersum et al. (2003).

Many factors affect crop growth and yield in agricultural fields and pastures. One innovation of early crop modeling pioneers was to categorize the crop production situation being modeled to narrow down the many factors that are needed by crop models (Bouman et al., 1996, van Ittersum et al., 2003). Fig. 1 summarizes three crop production levels and factors that influence each. Potential production is defined as crop production that is determined completely by defining factors of CO_2_, radiation, temperature, and crop characteristics (e.g., genetic control of physiology and canopy architecture). Potential production models also include partitioning of biomass growth into grain and other plant parts, with defining factors modeled to affect these processes. This potential production level is rarely achieved in real production situations, although under highly intensive management (supply of adequate water and nutrients and control of insects, weeds, and diseases), production approximates the potential level for the specific CO_2_, temperature, radiation, genetics, and canopy architecture used. For example, crops grown in greenhouses or in intensively managed fields in some regions produce yields that are at or near potential levels.

The next production situation is referred to as water-limited and/or nutrient-limited production (Fig. 1). At this level, the defining factors are still important, but there may also be limitations in the water and/or nutrients needed to achieve full growth potential. Crop models that simulate water and/or nutrient-limitations must include soil water and nutrient component modules to simulate the time-varying availability of water and nutrients, the uptake of these resources, and reductions in growth and development if they are not adequate to meet potential growth demands. Most cropping and grassland system models contain component modules that simulate soil water, nitrogen, and carbon dynamics because of the global importance of these resources in determining yield. Although some models include phosphorus, most of them do not simulate responses to phosphorus, potassium, or micro-nutrients. Models that represent soil water, nitrogen and carbon dynamics are complicated not only because of the physical and chemical processes that occur in soils, but also because of the complexities in management practices used for these resources (e.g., water-harvesting, drip irrigation, types of inorganic or organic fertilizer applied, fertilizer micro-dosing, etc.).

Finally, actual production (Fig. 1) includes additional factors that may reduce growth and yield (insects, diseases, weeds, and pollutants). Whereas some crop models have capabilities to introduce damage by diseases and insects (e.g., Boote et al., 1983, Pinnschmidt et al., 1995), the modeling of these reducing factors has not kept up with other advances in crop modeling (see below and Donatelli et al., this issue) for a review of recent progress). Most groups modeling cropping and grassland systems do not include these factors. Thus, few current models simulate responses to pest or disease damage or to their management using resistant varieties, agro-chemicals, or other approaches. This is a major limitation for some applications.

#### 2.1.3 Components of cropping system models – crop, soil, atmosphere, management

**Fig. 2:**
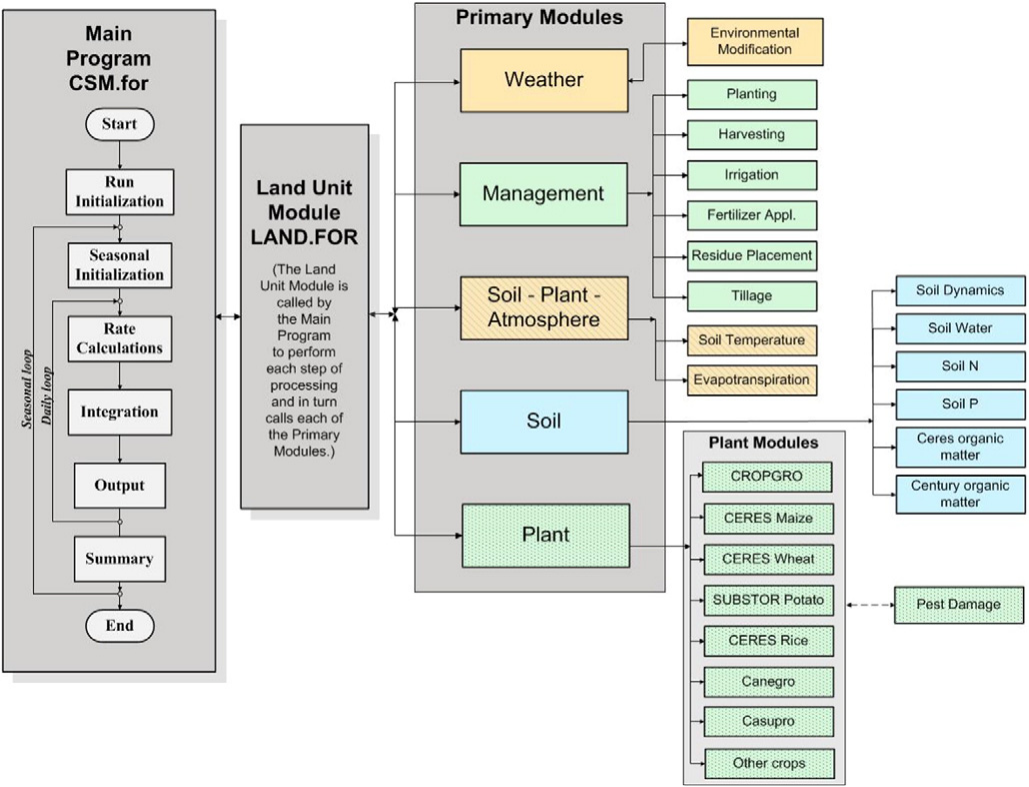
Land, soil, crop, climate, and management components in the DSSAT Cropping System Model.

Dynamic crop models generally include factors at the potential yield level (shown in Fig. 1) in addition to water- and nitrogen-limited production level. However, the ways that different models include those factors vary. Fig. 2 shows a schematic of the components in the Cropping System Model (CSM) that incorporates the CERES (e.g., see Basso et al., 2016), CROPGRO, and other models in DSSAT (Jones et al., 2003, Boote et al., 2010, Hoogenboom et al., 2015). The CSM models can include soil water, nitrogen, carbon, and phosphorus dynamics and can introduce pest and disease damage into some crops using the concept of coupling points (Boote et al., 1983, Batchelor et al., 1993). It also can simulate multiple seasons so that carry-over changes in soil water, N, and P are simulated to represent longer-term changes in soil resources in response to different management systems (Porter et al., 2010, Basso et al., 2011).

(CSM, adapted from Jones et al., 2003).

A number of other cropping and grassland system models have similar components and capabilities (e.g., APSIM, Keating et al., 2003a, Keating et al., 2003b and Holzworth et al., 2014; CROPSYST, Stöckle et al., 2003, Stöckle et al., 2014; EPIC, Williams et al., 1989; STICS, Brisson et al., 2003 and Bergez et al., 2014; SALUS, Basso and Ritchie, 2015 and Dzotsi et al., 2013), although most models do not simulate impacts of pests and diseases unless coupled externally with time-series input data or pest models like in DSSAT CSM (Boote et al., 1983, Batchelor et al., 1993). Some models (e.g., APSIM) have an ability to simulate intercropping (Thorburn et al., 2014, Holzworth et al., 2014). An unfortunate feature of current crop and grassland models is that modules from one set of models are not compatible with other models. For example, APSIM's intercropping capabilities are deeply embedded in the system architecture and cannot be simply moved to other models like DSSAT CSM. Moving pest and disease damage modules from DSSAT CSM to APSIM is possible but requires coding of module “wrappers” to handle inter-model communications – a non-trivial task.

#### 2.1.4 Approaches

Most “cropping system” models have evolved as elaborations of component crop and soil models and the focus has been on modeling a single “point” in space (consider this a field or a paddock) over time to explore variability in crop responses to soil, management and weather. A typical structure for this pedigree is shown in Fig. 2. Most operate on daily or hourly time-steps. Some include hourly time steps for computing rates of photosynthesis and other processes but also use daily steps to update state variables such as phenological development, and biomass of plant organs. These time steps are also used to compute changes in soil water, soil nitrogen, and crop biomass that result from soil-water processes including rainfall, infiltration, runoff, percolation, redistribution, and plant uptake, and changes in soil nitrogen. Details of how different growth, hydrology, and soil nutrient processes are represented vary among models.

Models may be either functional or mechanistic, with the choice of approach depending on the modeling team's knowledge of the system, their purpose, the availability of data for parameterization, and their experience in developing and evaluating models. These differences lead to different models producing different responses when used to simulate the same experiment (e.g., see Asseng et al., 2013, Bassu et al., 2014). Most models use simplified functional equations and logic to partition simulated biomass into various plant organs. Functional models also primarily use “capacity” concepts to describe the amount of water stored in a soil that is available to plants; mechanistic models, in contrast, use the potential energy of soil water and “instantaneous rate” concepts from soil physics. In capacity-based functional models, it is the difference between the upper and lower limits of soil water-holding capacity that determine the amount of water available to plants. In this type of soil water model, water movement and its availability for crop growth are represented by functional equations on a daily time step, even though infiltration and runoff processes may be computed with smaller time steps. Some modeling systems can operate with either capacity based or energy based soil water modules (e.g. APSIM) and ideally a flexible agro-ecosystem simulation engine or platform will be able to work with component modules specified to different degrees of “mechanism”. Although some models include input information on plant genetics (e.g., White and Hoogenboom, 1996, Hammer et al., 2006, Messina et al., 2006), these are few in number and not yet in widespread use. Most models are not genetic-based, which is one reason that calibration of models using field data is widely practiced to obtain genotype-specific parameters.

**Fig. 3:**
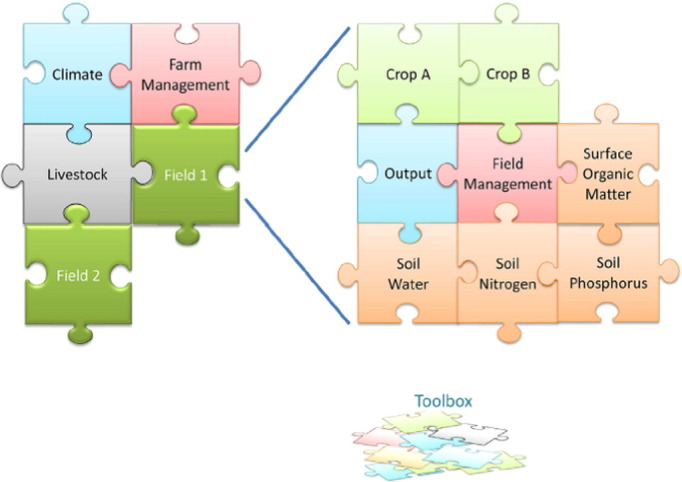
A conceptual architecture for “agricultural systems” simulation.

Some modeling platforms while utilizing crop and soil components such as shown in Fig. 2, have focused more strongly on “agricultural system” features, with capabilities of instantiation that facilitates the simulation of systems features such as multiple paddocks, intercropping, weeds, tree – crop interactions, livestock operations and even non-biological features of farms such as water storage structures. APSIM (Keating et al., 2003a, Keating et al., 2003b) is the best known example of this farming systems “platform”. It sits at the interface of the crop-soil systems models typified by Fig. 2 and the whole farm optimization models discussed elsewhere in this paper. Holzworth et al. (2014) outlines in full these “agricultural systems” features of the APSIM approach (Fig. 3).

(From Holzworth et al., 2014).

#### 2.1.5 Additional considerations for modeling grasslands

Grasslands are usually mixed stands comprised of a variety of grasses and forbs, including legumes and sometimes woody species (Allen et al., 2011). Unlike croplands, the diversity of species generally precludes use of a single-species parameterization, since species vary in their ability to compete for space, water, nutrients (most commonly nitrogen), and light. Grassland models generally represent plant behavior and competition among herbaceous plants using one of: (1) a set of species, each independently parameterized; (2) amalgamations of plants into parameters for plant functional types (e.g., warm-season grasses, legumes, etc.); or (3) community-averaged parameterizations (Taubert et al., 2012). While requiring more effort for parameterization, these amalgamated approaches enable representation of changes in plant community composition over time, for example in response to climate change, competition among plant populations, and mortality.

Trees are dynamic components of the world's native grazing lands and can have significant impacts on ecosystem function (Schlesinger et al., 1990). Representing tree/grass competition is challenging because trees respond differently to various drivers (such as fire, grazing, and CO_2_ concentration) and depend on plant population characteristics (e.g., seed banks). Shifts in plant community composition can be self-reinforcing due to co-occurring population and biophysical changes (D'Odorico et al., 2012). Dynamic vegetation modeling approaches are used to represent competition among herbaceous and woody types for water, nitrogen, light, and space. Dynamic rangeland vegetation models and state-and-transition models identify a set of plant communities that tend to resist change due to disturbance, but also describe drivers (e.g., fire, grazing, climate change) that lead to a transition to another quasi-stable plant community (Stringham et al., 2003).

Expansion of woody species and increases in woody cover are widespread phenomena that under many but not all environmental conditions lead to the transition of early successional communities dominated by grasses and forbs to forests (Van Auken, 2000). Studying woody encroachment and understanding the importance of competing drivers has been challenging, in part because of the slow rates of the processes driving changes (e.g., Morgan et al., 2007). These slow changes are reflected in the drivers of transitions in state-and-transition models and contribute to uncertainty in our ability to represent longer-term changes in the tree-grass balance. Ecological succession has been studied by plant ecologists since pioneering work before 1945. More interactions among agricultural and ecological modelers are likely to be mutually beneficial.

Grazing animals of all kinds have an impact on plant productivity by removing photosynthesizing tissues, altering light transmission through the canopy, influencing nutrient cycling and affecting plant allocation patterns and differentially influencing species mortality and recruitment rates in grasslands (Diaz et al., 2007). Such changes to groups of plants (species, functional groups, etc.) can drive changes in the competitive balance and thus plant community composition. Whereas grassland models incorporating species or plant functional types can represent grazing-induced changes in the competitive balance, such models that represent plants with a set of community-wide parameters usually rely on some combination of LAI (Leaf Area Index)-driven reduction in production potential along with grazing response curves. In grasslands/rangelands, grazing (or cutting for hay) removes some plant productive capacity, and thus models cannot rely upon deterministic growth curves, but must be able to forecast growth for plants with an amount of biomass or leaf area that varies independent of the time of year or climate. There can also be significant differences in growth rates among and even within species after a grazing event (Milchunas and Lauenroth, 1993, Vesk and Westoby, 2001).

### 2.2 Reduced form summary crop models

The factors to which models respond vary among models and evolve as modelers attempt to make them more comprehensive and universally applicable. In contrast, some researchers who want to apply them do not have all needed inputs, or they may want to embed a crop model into economic or other models for analyzing responses across scales. Some researchers have used more comprehensive crop models to create reduced form crop models that have much fewer input requirements, run fast, and produce responses needed for specific applications (e.g., Jones et al., 1999). For example, Chikowo et al. (2008) used the APSIM cropping system model to generate parameters and variables needed to operate a much simpler field scale crop model (NUANCES-FIELD, Tittonell et al., 2007). The reduced form summary model responds to nitrogen and phosphorus levels for different soil characteristics and management inputs. Dzotsi et al. (2013) used a similar approach, showing that reduced maize, peanut, and cotton models parameterized from the DSSAT CSM model accurately reproduced DSSAT results across time and space.

Reduced form crop models allow researchers to produce situation-specific summary models that approximate the responses of a more comprehensive model for use in broader scale analyses that may involve socioeconomic, livestock, and environmental sustainability components. Reduced-form crop models can be interpreted as the “production function” that is the foundation of economic production models (see Section 2.5), and can be linked to economic models to create “hybrid” models for policy analysis and impact assessment (Antle and Capalbo, 2001, Antle and Stockle, 2015). Keating et al., 2003a, Keating et al., 2003b demonstrated a similar process of summary model development, building from the foundation of a comprehensive set of crop-soil and management system simulations. They developed a summary model from thousands of APSIM simulations with three key parameters that captured 88% of the variation in space and time of key water balance variables.

### 2.3 Livestock systems

Livestock systems are complex and require modeling at several levels: the animal, the herd, and its interactions of the herd with its environment via consumption of feed, use of land and water, and other resources. Several types of models have been used in the past to describe different components of livestock systems. (see reviews by Herrero et al., 1998 and Tedeschi et al., 2014). Examples of these are DSSAT (Jones, 1993), APSIM (Holsworth et al., 2014), Century (Parton et al., 1993), SAVANNA (Coughenhour, 1992), The Hurley Pasture Model (Thornley, 1997), PHYGROW (Stuth et al., 2003). Table 1 summarizes the most commonly used types.

**Table 1:** Livestock models and some types of questions they can help answer.

Type of model	Simulation	Outputs
Individual animal performance	-Prediction of performance Assessing impacts of alternative feeding practices on yields, GHG emissions Assessing sustainable intensification strategies Impacts of changes in breeds or types of animals -Yield gap studies Assessment of manure quantity and quality Establishment of substitution rates between feeds -Impacts of feed scarcity	-Least-cost diet formulation -Optimization of supplementation practices -Nutrient synchrony studies -Amino acid adequacy for high yielding dairy cows Optimal feed management for different types of animals in a herd
Herd dynamics	-Impacts of reproductive management -Stocking rate decisions Impacts of climate variability on herd dynamics Epidemiological studies of disease spread and impacts on herd numbers, profitability -Value chain studies	-Optimal replacement -Optimal times to sell animals -Impacts of climate variability on herd dynamics
Integrated livestock systems	Assessment of the feasibility of new management strategies -Land use management strategies -Best grazing practices -Feed conservation strategies Trade-offs in the use of resources technology targeting Identification of key constraints (labor, etc.) -Gender sensitive strategies -Selling and replacement strategies	-Optimal herd sizes -Land-use management in livestock farms -Intensification potentials -Trade-offs in the use of resources Impacts of input and output prices -Impacts of intensification or environmental policies Optimal stocking rates and carrying capacities -Selling and replacement strategies Matching seasonal feed resources to herd dynamics

#### 2.3.1 Animal performance models

A central element driving production, profitability, and efficiency in livestock systems is animal performance. Hence, the most commonly used livestock models are those that predict animal meat and milk productivity. Precursors to performance models have existed since the 1940s when the first feed requirements for livestock were developed (NRC, 1945). Since then, many have been built and refined regularly across the US and Europe (AFRC, 1993, NRC, 2001). Nutrient requirements models are the workhorse of the feed industry for ration formulation (linked to linear programming models for least-cost ration formulation) and for recommending changes in feed management to farm advisors. These models are often based on a mixture of statistical regressions derived from experimental data plus mechanistic principles of the energetics and protein metabolism of mammals.

Animal performance models usually require information on the animal (i.e., bodyweight, target milk production, milk composition, breed, days pregnant) and the feeds (digestibility and crude protein at the minimum, but increasingly several parameters related to the fiber, mineral and/or amino acid composition of feeds are also used). They also need an estimate of feed intake, perhaps the most important parameter. While these models are good for calculating feed requirements, dynamic models of digestion are more accurate at predicting the nutrient supply to animals under a wide range of conditions from the high-yielding dairy cow to the smallholder goat (Tedeschi et al., 2014, Herrero et al., 2013, Illius and Allen, 1994), because they predict intake more accurately, and they can deal with more complex diets and their interactions.

Some models also predict methane production by ruminants and manure quantity and quality, which are important for estimating GHG (greenhouse gas) emissions and the role of livestock in nutrient cycles. These models are typically used to answer ‘what if’ questions around the impacts of different feeding practices (different feeds and/or different quantities) or changes of animal types (breeds, different production potential) on animal performance (meat and/or milk output, GHG emissions, manure output).

*Herd dynamics models* follow herd evolution over time in terms of animal numbers and herd structure. Herd dynamics models usually start by splitting a herd into cohorts of different ages or weight, and sex. These cohorts are specified with different mortality, reproductive, selling and replacement rates. Adult females produce offspring at specified reproductive rates, which grow or die, become part of the next cohort, and get sold, and the cycle continues.

The best of these models include interactions between animal nutrition and reproduction to drive reproductive and mortality parameters stochastically (Konandreas and Anderson, 1982, Rufino et al., 2009). This feature is important as feed availability or supplementation strategies have significant impacts on herd reproduction and performance. Some applications of herd dynamics models include estimating optimal stocking rates and carrying capacities, assessing the impacts of reproductive technologies and/or reductions in mortality, and predicting removal of biomass from crop or pasture systems. These models are also widely used by livestock epidemiologists for estimating impacts of diseases on herd mortality and morbidity. They have also been used with dynamic programming for optimizing replacement decisions in commercial dairy herds (Van Arendonk and Dijkhuizen, 1985), or in linear programming applications for studying optimal sales policies, herd sizes, etc. Biological simulation models are sometimes used as input-output coefficient generators for linear programming models to aid in the selection of management strategies in livestock systems (Woodward, 1998, Nicholson et al., 1994, Herrero et al., 1999).

#### 2.3.2 Integrated livestock systems models

**Fig. 4:**
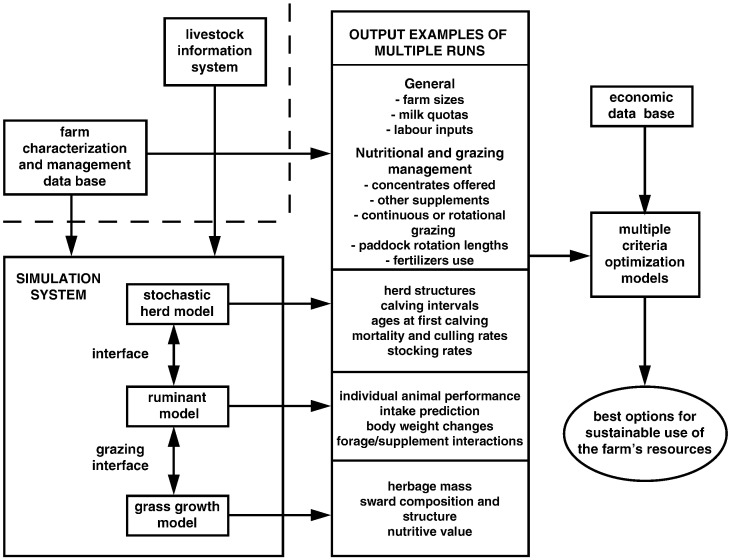
Integrated livestock modeling framework.

These models represent whole livestock farms and their key components (Fig. 4). The complexity of some livestock systems justifies the need to build whole-system models using simulation and optimization techniques to represent different components and their interactions (Herrero et al., 1996, Herrero et al., 2007, Thornton and Herrero, 2001). For example, grazing management strategies cannot be defined without also considering herd and nutritional management, since herd dynamics or feed supplementation practices determine the grazing intensity, use of forage, and subsequently animal performance. Thus, simulations of the biology of livestock enterprises include flexible models representing pasture growth, structure and quality; individual animal performance to test nutritional strategies; and population dynamics describing management practices at herd or flock level (i.e., stocking rates; sales of animals; mortality or replacement rates; calving intervals), which subsequently determine animal numbers and their age or physiological state (lactating vs. pregnant cows, heifers, calves, etc.) classes (Freer et al., 1970, Freer et al., 1997, Loewer, 1998, Johnson, 2002).

(Adapted from Herrero et al., 1996).

### 2.4 Modeling pests and diseases of crops and livestock

Biologists have been building mathematical models to describe the population dynamics of agricultural weeds, pests and diseases for more than a hundred years. Recent progress in modeling these components is discussed by Donatelli et al. (this issue); here we focus more on broad concepts and general state of progress. The diversity of modeling approaches that constitute the current state of science can be categorized in different ways. The first and most obvious is by production type and threat. Thus there are models that describe the dynamics of weeds, diseases and pests that are threats to arable crops, the diseases of livestock, and the diseases of fish used in aquaculture. While threats such as pests and diseases have been recognized since pre-history, the complexities of the microbial communities on the crop surface and in the soil around plants, and in the gut and rumen, are only just becoming more fully understood. Models of the mixture of beneficial and pathogenic organisms that these systems contain have not yet been developed.

A broad distinction can be made between mechanistic (or process-based) and non-process-based pest and disease models. The former include explicit biology while the latter use a purely statistical approach. The choice of modeling approach depends greatly on the intended application. For example, a farm manager may want to know when to apply a prophylactic insecticide against a common insect pest. For this purpose, future insect population density may be best predicted by a statistical model containing independent biological variables such as crop stage, and dependent weather variables such as temperature and rainfall. In some cases, information about the pest itself may be included in the model, for example from pheromone or other traps monitored by the farmer or in the case of mobile insects from publicly-operated monitoring networks.

A different statistical application is the use of climate-matching models to predict future pest problems. The current distribution of an organism is modeled using a set of predictors including climate. The distribution of the organism after climate change is then estimated by mapping the “climate envelope” using scenarios developed from global climate models. There is now a broad literature on the strengths and weaknesses of this approach, particularly challenging the assumption that organisms are able to move easily to track climate. Important recent advances in statistical models of pest dynamics have included the application of modern spline and neural net estimation techniques, and in the use of personal computers and mobile devices.

Mechanistic models incorporate at least some information about the biology of the crop and pest species concerned. The models may be highly abstract – summarizing, for example, a pest population by a single state variable such as density – or, alternatively, highly complex with individual pests each represented by numerous attributes. The simplest models sacrifice realism for mathematical tractability and general insights, while models of intermediate complexity include more biological detail but are constructed in such a way that simpler analytical models can be recovered as limiting cases to help interpretation. Pest and disease models also vary in the degree to which they explicitly incorporate stochastic processes (often critical in epidemiological models) and in whether they treat a population as homogeneous or spatially variable.

An important area is the coupling of pest and disease models with crop models (Boote et al., 1983, Willocquet et al., 2004, Savary et al., 2006, Whish et al., 2015). Donatelli et al. (this issue) review issues involved and existing major projects that have attempted to bridge this gap. They also propose a roadmap to improve pest and disease modeling focusing on improving the data resources available for parameterization and validation, bettering the coupling of crop to antagonist models, and creating a community of researchers that can collaborate to share expertise and produce community tools.

#### 2.4.1 Near-future pest and disease threats

Mechanistic models can be used to predict near-future pest and disease threats in similar ways to statistical models. As was discussed with crop models, they may be more successful than statistical models if biological insights can substitute for missing data or if they can aid prediction by suggesting a model structure that simple statistical fitting would miss. Consider, for example, the response of an insect to daily temperature. Higher temperatures may elevate growth and reproduction, and thus result in more pests, a pattern that could be derived with sufficient weather and population data. Alternatively, the physiological response of the insect could be modeled, which might improve the model's predictive power or allow insect dynamics to be predicted in data-poor systems (or under future climate). Several schools of physiological modeling exist. However, we are not aware of any formal comparison of different process and statistical approaches to the same problem.

An area where biological insights have proven fruitful has been in disease spread through commercial livestock populations. An understanding of how animals interact, and more importantly how they are moved around, can provide critical advice to policy-makers. Current state-of-the-art livestock models incorporate data on movements of animals between individual farms coupled with modern Bayesian parameter estimation. However, the type of data needed for such approaches is prohibitively expensive to obtain or politically unacceptable for many countries to collect (Brooks-Pollock et al., 2014).

Modeling has also proved valuable in assessing possible pest risks and in guiding general policy development. The basic epidemiological number (R_0_) is the number of secondary cases of a disease that are expected to happen when a primary case occurs in a susceptible population. Calculation of R_0_ for prevalent human diseases has proved useful in prioritizing investment in control strategies and vaccine development. Today, sophisticated mathematical tools are available for calculating R_0_ for complex structured populations, for spatially extended populations, and in the presence of stochastic effects (Diekman et al., 2012).

Probably the most sophisticated applications of population genetics to weed, pest and disease issues in agriculture are models of the evolution of resistance to pesticides, and of the dynamics of plant diseases. Evolutionary models can be broadly categorized as genetic or phenotypic. Although phenotypic models have been explored in agriculture (e.g., Denisen, 2012), the vast majority of evolutionary models have been genetic. Based on theoretical analyses, areas of fields have been set aside unsprayed or not planted with modified crops that express an insecticide in order to slow the rate of spread of resistance (Bates et al., 2005). The genetic basis of plant-pathogen interactions have been resolved for a number of major systems, which has allowed detailed analysis of strain dynamics and how disease spread may be slowed by judicious use of a range of different crop varieties. State-of-the-art work in genetic models of weeds, pests and diseases includes using the avalanche of data that modern high-throughput DNA measurement technologies are providing, and modeling how novel genetic interventions may be used to suppress pest populations. Some of the most sophisticated pest monitoring software (typically based on statistical rather than on process models) now includes specific economic variables with parameters such as commodity prices that can be updated dynamically. The farmer may make different decisions about pest management depending on current market conditions.

More generally, a goal of many people working to increase the sustainability of agriculture is to reduce chemical inputs by practicing “integrated pest (or disease) management”. The models required to support such work are challenging to construct, but some of the most advanced incorporate economic elements as well as various biological processes.

### 2.5 Economic models

A number of approaches have been developed to model the economic implications of decisions and policies for a range of scales and purposes. Here, we summarize the most important approaches that have been developed and present important limitations of each.

#### 2.5.1 Farm management linear programming models

Linear economic optimization models of farm systems, developed in the 1950–60s, provide a basis for prescriptive farm management advice (Heady and Dillon, 1964). These models are characterized by a complex set of linear inequality constraints that represent the production possibilities available to a farmer. The simplex optimization algorithm is used to select the optimum production possibilities. One disadvantage of this approach is that the solutions are restricted to extreme points in the multidimensional decision variable space and thus it is unable to explore intermediate solutions. A major problem with linear programming models is that they need complex constraint structures to achieve some degree of calibration to base data; those constraint structures restrict alternative solutions and are difficult to implement for applications such as adoption and impact of new technologies.

#### 2.5.2 Econometric production models

Econometric methods have been developed and used for single crop production function models as well as single-equation and simultaneous system models that represent input demand and output supply behavior. Early work focused on primal representations and statistical estimation (Mundlak, 1961), but many efforts shifted to dual representations in the 1970s and later (Lau and Yotopolous, 1971, Chambers, 1988). Both static and dynamic models have been developed. Single crop production functions are estimated directly from data on the physical quantities of inputs and outputs observed from experimental plots, or, in later stages, from comprehensive farm production surveys. Heady (1957) was an early proponent and researcher in this area. In many cases the functional form for the production functions is a quadratic or Cobb Douglas specification, both of which have implicit restrictive assumptions on the production technology. Later work emphasized various more flexible technology representations (Carter, 1984).

Econometric estimation of agricultural systems was expanded to represent both multi-crop production with its associated interdependencies, the endogenous nature of agricultural supply response, and the imputed value of some key agricultural inputs that are often incompletely priced. A landmark article in this literature (Just et al., 1983) noted that multi-crop farm businesses responded to changes in prices or technology by adjusting both the intensity of input use per acre, the intensive margin (i.e., fertilizer amount per land area); and also the allocation of land to crops, the extensive margin. This distinction is important for modeling optimal input allocation in multi-crop farming systems. The importance of the interaction of multi-crops in a farm unit was a significant step forward in realistic economic models of farming systems. However, the approach did not include formal linkage to biophysical models of agricultural processes. The econometric approach has limitations in its ability to extrapolate responses that are outside the estimation sample, or those that employ systems that are not present in the data sample. These limitations were emphasized by Antle and Capalbo (2001) in their development of economic simulation models that combine econometric and other disciplinary simulation models into an integrated assessment framework.

#### 2.5.3 Risk behavior models

The importance of risk on farm decisions was recognized early in the development of linear optimization models of farming systems. Early articles on this linear approach to risk analysis are by Lin et al., 1974, Hazell and Scandizzo, 1975. As improved algorithms to solve quadratic optimization problems were developed, specification of risk expanded to a mean-variance measure of risk and imputed a risk-aversion value based on observed farmer actions or primary surveys (see Hazell and Norton, 1986). Just and Pope (1978) introduced a widely-used econometric risk model. Antle (1983) introduced a general moment-based representation of output distributions that has been widely used to study production risk behavior, including downside risk. Recent research has extended this approach to investigate impacts of climate change (Tack et al., 2012).

#### 2.5.4 Spatial equilibrium models

The importance of space in agricultural production and modeling agricultural systems was first introduced in terms of trade between regions of different comparative advantages. Takayama and Judge (1964) showed that spatial equilibrium conditions and transport cost between different production locations could be characterized as a quadratic optimization problem. Spatial econometrics advanced to include rates of development and specialization of production (Anselin, 1988). Only recently has the availability of remotely sensed measures of agricultural land and water use led to the use of spatial econometrics methods to address spatially varying farm production (Anselin et al., 2004, Staal S. et al., 2002). Techniques are emerging that use both remotely sensed data and spatial econometrics to draw conclusions about resource use or the effect of spatial variation on agricultural supply response.

#### 2.5.5 Structural simulation models

Complex simulation models have been used for the past 45 years to describe dynamic agricultural systems. Early examples were often based on Forrester's (1968) concept of system dynamics that uses storage and flow variables to describe the system. However the underlying philosophy that a comprehensive and complex feedback system is stable and reproducible has never been convincingly demonstrated. Structural simulation models can be useful for representing a combination of consistent behavioral relationships (i.e., that the quantities of product supplied by farmers can be sold at a price that recovers the costs of production inputs) based on theory and empirical measurement. They are however, subject to interpretation in the absence of robustly estimated relationships describing system behavior.

Various micro-economic models have been developed to simulate the economic behavior of agricultural systems and link behavior to environmental processes and economic sustainability indicators. van Wijk et al. (2014) document the large number and diversity of such models, that include: applications of various types of linear or non-linear programming models; household models (i.e., models that combine production systems with household behavior such as food consumption and labor supply); agent-based models that incorporate spatial and temporal interactions among households; and models that link economic models with bio-physical crop, livestock, and environmental models. Recently, agent-based modeling (Billari et al., 2006, Berger and Troost, 2014) has been widely used as a way of modeling interactive human behavior and natural systems. Some agent-based models have a more formal dynamic and calibration structure and use mixed-integer optimization approaches for solutions. However, the generality of the approach makes it susceptible to the same difficulties of empirical verification and reproducibility that earlier complex structural simulation models had. The population-based modeling approach of Antle et al. (2014) is a more parsimonious, generic approach designed to represent agricultural system heterogeneity. It links economic simulation models to bio-physical models to evaluate impacts of technology, policy and environmental changes on sustainability.

#### 2.5.6 Calibrating optimization models

Along with more complex constrained models, researchers have developed optimization models that utilize shadow values of resources and calibration constraints to derive nonlinear calibrating functions, which are termed positive mathematical programming (PMP, Howitt, 1995). In the past 10 years PMP has developed from formal calibration methods that reproduce the observed cropping pattern (first-order calibration) to those that calibrate crop supplies to prior estimates of supply elasticities (second-order calibration), and more complex production functions that calibrate against elasticities of substitution and returns to scale. In addition, PMP models are now being formally linked with biophysical models (see Mérel and Howitt, 2014 for a survey of recent developments).

#### 2.5.7 Computable general equilibrium models

These macroeconomic models (e.g., computable general equilibrium, or CGE models) spawned a series of smaller-scale models which are usually called village or household models. General equilibrium village models account for all flows in the village economy and remittances within the village to different workers and landowners. In addition, they include flows of revenue in and out of the village boundary. This is particularly useful in developing country farm economies where much of the labor is supplied by family members with little or no pay. Another advantage of village-level equilibrium models is that they account for the utility gained from subsistence food grown in a village. These CGE models are anchored by a social accounting matrix that accounts for flows within and outside the economy. Moreover, it is common practice to fit the standard functional form such as a constant elasticity of substitution production, supply, or transformation function that is calibrated against exogenously estimated elasticities (Taylor and Adelman, 2006). CGE models have the disadvantage of being data and computationally intensive due to their more general specification, and for the quite restrictive assumptions required for their solution. Compared with more detailed partial equilibrium models, general equilibrium models are harder to incorporate detailed process models.

#### 2.5.8 Integrated bio-economic models

**Fig. 5:**
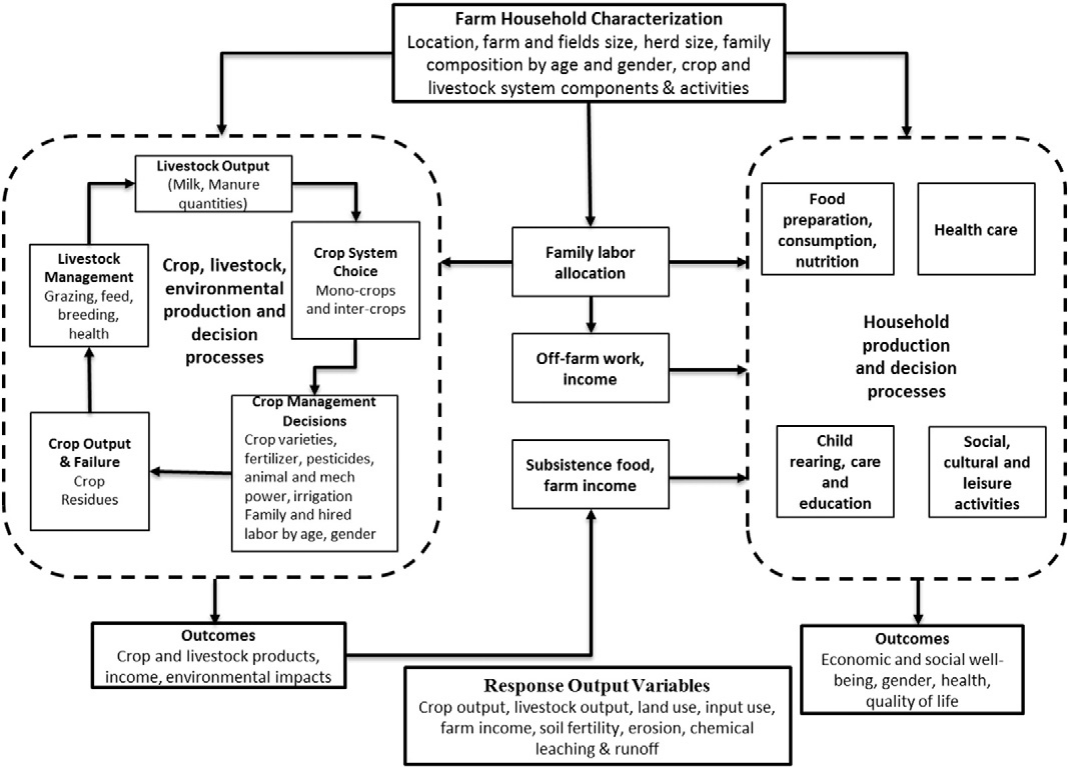
Diagram of a farming system showing the household and production system components and interactions that need to be included in models.

Flichman (2012) describes recent studies on application of models that combine bio-physical and economic models to represent agricultural systems. Flichman and Allen (2012) and van Wijk et al., 2009, van Wijk et al., 2014 also survey economic agricultural system models. They characterize bio-economic models into farm, landscape, regional, and national models. Systems in each of these scales include crops, livestock, and socioeconomics components that interact in complex ways. For example, Fig. 5 shows components that need to be included in system models at the farm scale. These components and processes encompass the crop and livestock production enterprises of a farm, the household decision and production processes, and the interactions among the household and production systems of the farm.

Within these scales the cited authors address both static and dynamic specifications. In his introduction Flichman (2012) attributes growth of bio-economic modeling to two developments: improvement of biophysical agricultural simulation models, and evolution of agricultural policies that demand integrated assessments that conventional economic models cannot provide. We briefly address three prominent areas of application of integrated bio-economic models.

i. *Climate Change Impact Assessment Models.* Economic modeling approaches on impact of climate change on agricultural systems have been addressed in very different ways. The first method links optimization models of agricultural production to climate models using agronomic models which map climate change variables into crop yields effects. An early example of this type of modeling on a national basis for the United States is by Adams et al. (1990); results from this type of model were widely used (e.g., IPCC, 1990, Rosenzweig and Parry, 1994). Since then, this same approach has been used in different types of optimization models over smaller geographic areas and driven by downscaled climate data. One advantage of using formal economic models to estimate impacts of climate change is that they can incorporate effects of both adaptation and mitigation that result from economically optimal adjustments to agricultural production under both changed growing conditions and a carbon tax.

An alternative econometric approach to measuring the impact of climate change both on agricultural crop yields and on economic variables such as land values and economic returns is to estimate statistical models based on observed behavior. These statistical models are then simulated with data from future climate projections. A justification for this approach is that it can embed realistic adaptive behavior into the model (Mendelsohn et al., 1994). However, this type of model also has significant weaknesses. For example, it does not incorporate effects of CO_2_ fertilization on crop productivity, cannot represent changes in socio-economic conditions, and cannot be used to identify technological adaptations distinct from climate impacts. Various researchers have used statistical econometric methods to model the effects of climate on yields and other variables (Schlenker et al., 2013).

ii. *Hydro-Agricultural Economic System Models.* There is a long history of modeling hydro-economics of agricultural systems, since irrigated agriculture is the largest user of water in many parts of the world. The tradition of integrating hydrology models with economic models stretches back 35 years since it was recognized early that the motivation for water use is strictly economic in agriculture, but that the equations associated with water use had to be modeled by physical hydrologic models. Accordingly, hydro-economic models were developed as coupled individual modeling systems. Two approaches are used. In the first one, economic models provide benefit-response functions, which are then embedded in a hydrologic policy model (e.g., the Calvin water allocation model, Jenkins et al., 2004). A second hydro-economic modeling approach is to characterize the response in the hydrologic models by statistically fitting a simplified function to results from complex simulations over a range of hydrologic and climatological parameters. These response functions can then be included in an economic policy model. This approach has often been used to analyze the optimal management of common property resources used in agriculture, such as groundwater (e.g., Knapp and Olson, 1996). A review of concepts and applications of hydro-economic models from an economic perspective can be found in Booker et al. (2012), while Harou et al. (2009) published a similar survey from a hydrologic perspective.

iii. *Integrated Economic Livestock Models.* These models usually fall into one of two combinations; namely biological process models with an economic component, or an economic model with livestock equations and response functions. Several models in developing countries take into account household linkages and village-level interactions where there is some degree of subsistence consumption of livestock products, most commonly milk.

Havlik et al. (2014) provides an overview of integrated livestock modeling and its use in mitigating climate change. Their analysis is driven by a large-scale economic optimization model that assesses crop bioenergy production, land-use changes, water requirements, and greenhouse gas emissions. Their results show that improvements in livestock production systems can significantly reduce impacts on fragile land use and improve the effectiveness of climate mitigation policies. In another approach, Kobayashi et al. (2007) analyzed stocking density impacts on Kazakhstan's extensive rangelands using a stochastic dynamic programming model for multiple livestock systems with stochastic forage production. They showed that cost of capital strongly affects herd size and productivity.

### 2.6 Landscape/watershed; water and environmental quality

There is a rich history of modeling watershed and environmental quality, however much of this has not incorporated goals related to agricultural systems and only a few efforts incorporate crop and livestock models. There are at least two different perspectives about modeling across space, including the interconnectedness of agricultural and ecological systems across the landscape. The first perspective is that human systems, including the farm, communities, and administrative and political areas in which agricultural systems interact through decisions and policies, affect production systems, markets, and trade. The other perspective is that the interconnectedness among hydrological and biophysical processes establishes the underlying behavior of agricultural systems over the landscape. This perspective leads to an emphasis on understanding physical, chemical, and biological processes that occur in watersheds. Both perspectives are important, yet agricultural models rarely consider both in the same assessments or models. There are many applications of watershed hydrology models, in particular using the SWAT model as discussed by Gassman et al. (2014), mainly focusing on environmental quality and water resource issues.

**Fig. 6:**
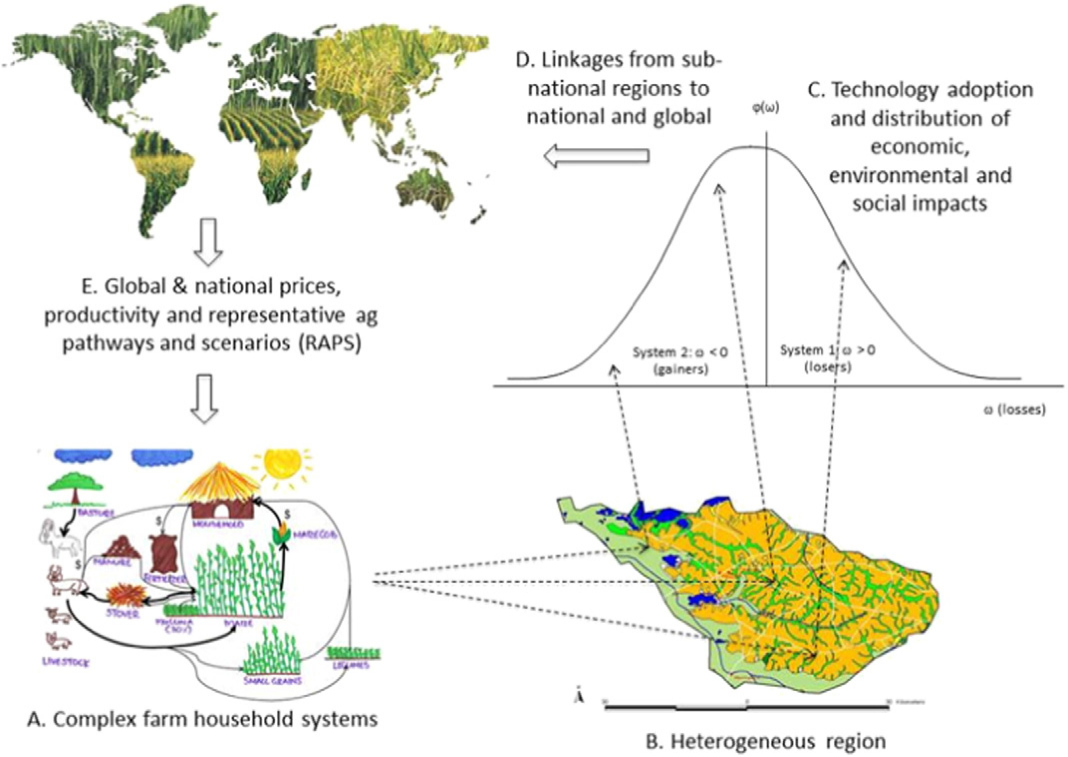
The AgMIP Regional Integrated Assessment framework emphasizing linkages across scales and analysis of distributional impacts in heterogeneous populations of farm households (Antle et al., 2014).

Fig. 6 shows the regional integrated assessment approach developed by AgMIP that emphasizes linkages of agricultural systems across space using the first perspective noted above (A. farm household; B. heterogeneous farms in one or more communities across the landscape; C. farm population heterogeneity; and D. national/global scale). In this perspective, based in part on the impact assessment approach developed by Antle (2011), the focus is on the economic, environmental, and social impacts of alternative systems within heterogeneous household populations. However, this framework also illustrates the feedbacks from farms to agro-ecological regions to national and global scales.

We often use the term “scaling up” of model results to refer to the aggregation of model results from finer spatial resolutions to a larger area. If the areas of interest are defined by hydrologists, they tend to be watersheds. In contrast, if the areas are defined by economists, they tend to be administrative and political units (e.g., urban areas, districts, countries) or socio-economic stratifications (e.g., small and large farms). These perspectives are not mutually exclusive, however. In fact, they lend themselves to include both human and biophysical/hydrological processes. A challenge for next generation agricultural models is to include the technical aspects of integrated modeling and a transdisciplinary approach in which scientists recognize the need for collaboration, not only on specific projects, but also in designing models and decision support tools to achieve their goals.

Many current agricultural system models have been developed to evaluate practices and policies associated with environmental quality. Biophysical models (e.g., crop or nutrient models) typically operate at the point/field scales with an emphasis on vertical fluxes of energy, water, C, N and nutrients throughout the atmosphere, plant, and soil root-zone continuum. Upscaling from point to the landscape scale requires estimation of surface and subsurface fluxes and ecological transitions along the lateral scale. Coupling with landscape microclimate models provides not only the vertical inputs used by agricultural models, but also gradients (precipitation, temperature, wind, vapor pressure deficit) along the landscape. Coupling with hydrological models provides water flow paths such as surface runoff, vertical and lateral groundwater flow, and interactions between shallow soil and groundwater zones and with adjacent surface water bodies (channels, rivers, lakes and coastal waters). Water quality models provide sediment and solute transport along the landscape controlled by water flows, and other effects such as wind erosion.

Integration and upscaling of landscapes into the watershed scale requires three-dimensional coupling of the surface and subsurface water, energy and mass transfers. Condon and Maxwell (2013) and Maxwell et al. (2014) provide more details on coupled versus integrated models. At this scale, the groundwater aquifer system typically transcends the boundaries of the watershed, necessitating regional scale analysis to evaluate not only the impacts of cropping and animal production systems on water quantity and quality, but also feedbacks from the hydrological system into the agricultural system (shallow water table effects and drought or low water availability for irrigation (Muñoz-Carpena et al., 2006). Further, meso-scale rainfall and evapotranspiration distribution models control the local surface and subsurface flow intensities, pollution and abatement (Shrivastava et al., 2014). At this scale, human effects through land use changes, and ecological (vegetation, wildlife) dynamics and transitions on natural or protected lands (riparian zones, conservation areas, etc.) are also important components needed to evaluate overall sustainability of agricultural systems (Matson et al., 1997). Although some efforts have gone into integrating biophysical models (e.g., crop, hydrology, livestock, ecological, and economic), more is needed to enable comprehensive assessments of agricultural systems across scales and adequately address environmental and economic responses to decisions and policies.

### 2.7 Aggregate agricultural system models (district, country)

The need to address decisions and policies at scales arises frequently in agricultural system modeling. Resolving the time and space scale differences among model components is often a major issue, particularly when component models are developed independently for different purposes. This problem arises, for example, when one attempts to create a model that combines crop and hydrology models, crop and economic models, or crop and climate models (Challinor et al., 2004, Osborne et al., 2007a, Elliott et al., 2014).

There have also been efforts, starting in the early 2000s, in which dynamic models have been developed to provide forecasts over aggregated areas (e.g., to provide aggregate crop forecasts). Traditionally, climate model output for a grid cell is downscaled to produce weather data time series for points that are then fed into crop models. However, the land surface also influences climate; processes within the atmosphere and oceans, and on the land, are coupled and dynamically interact over space on timescales from fractions of seconds to thousands of years. Crops are a major component of the land surface of the globe, occupying about a quarter of all land area. Regional climate can be sensitive to large-scale changes in cropped areas that can result from changes in economic or climate conditions (Osborne et al., 2004). Therefore, another direction for agricultural impact assessments at a large-scale is to dynamically couple crop simulation with models of land and atmospheric processes.

Five research groups have succeeded in coupling aggregate crop into climate models (Bondeau et al., 2007, Gervois et al., 2008, Kucharik, 2003, Osborne et al., 2007b and Stehfest et al., 2007). Osborne et al., 2009 showed that, in some parts of the world, the impact of changes in cropped area on regional surface temperature can be of the same magnitude as regional human-induced climate change This result raises the question of whether or not new fully-coupled climate change impacts studies will revise our previous estimates of food security impacts. It is clear that the full coupling of crop simulations within global climate models is opening up new possibilities for studies of the impact of climate change on agricultural production – studies that capture some of the complex and important feedbacks within the Earth system at a large scale.

Limitations in the skill of large-area modeling of crop production and yield is dominated by the density of data used in the simulation. More data should equate to better skill. However, the skill of large-scale modeling is determined by the smallest data set, whether this is the grid cell with the shortest run of observed yields, or the data grid with the largest resolution (climate, crop, soils or otherwise). We have seen recent increases in the resolution of climate input data and global grids of crop management and soil information. In this field of agricultural modeling, any future increase in data resolution should produce more skillful model simulations.

## 3 Current agricultural system models in context of selected use cases

We next discuss the state of current agricultural system science relative to its capabilities and limitations in providing information to assist a wide range of decision makers represented by the five Use Cases. Each Use Case contains a set of interactions between systems and users in a particular environment in a systems analysis. The Use Cases are for developing and developed country settings, demonstrating a range of needs for widely different applications at different scales and levels of intensification. Antle et al., 2016a, Antle et al., 2016b indicated that these Use Cases need crop, livestock, and farming system models. The question that we address here is whether current agricultural system models, existing sources of data, and existing decision support systems (DSS) are adequate for providing information needed for these Use Cases.

### 3.1 Farm extension in Africa

In this Use Case, the user is Sizani, an extension officer providing advice to a small farmer in Southern Africa, to help her and her family consistently produce more food and income.

#### 3.1.1 Capabilities and limitations

##### 3.1.1.1 Models

Can existing crop, livestock, and farming system models, data, and ICT tools provide the information that Sizani needs to advise the small farmer? The short answer is “No”; there are currently no easily accessible and usable applications that would allow her to analyze the particular farmer's situation. Or apps that can connect with models in the “cloud” to make runs needed for her to advise the farmer. Although there are models that partially meet her needs, and there are well documented examples of using models to develop insights on productivity enhancement strategies in the face of resource constraints and climate risk (e.g., Keating and McCown, 2001) they have not been integrated or are not packaged for use by this type of non-expert user. Models can, for example, simulate responses of crops to soil and weather conditions as well as water and nitrogen fertilizer input (Fig. 1) but do not generally simulate actual yield in production situations where, weeds, pests or diseases are not controlled.

Two of the most serious limitations of many crop-soil models are their inabilities to accurately simulate soil infertility and their failure to represent losses associated with the wide range of pest, disease, and weed species that damage crops. In many intensive production systems, soil fertility, weeds, pests, and diseases are controlled so that responses in those situations can be represented by the costs of management inputs and the production responses to climate and water management. Typically, cropping system models simulate yields that are higher than actual yields in farmers' fields, which are reduced due to poor management. In addition, fields are usually not homogeneous; for example, spacing between plants may vary considerably, whereas the models assume homogeneity. However, if pest and disease data are observed and available, these data can be input to some existing crop models to compute yield loss associated with specific pests and to diagnose the reasons for the gap between potential and actual yield (including the gap associated with water and some nutrients, especially nitrogen and phosphorus; e.g., Boote et al., 1983, Naab et al., 2004, Naab et al., 2015). Keating and McCown (2001) have shown, however, that expert application of well adapted models can still lead to useful insights on many of the key constraints to productivity enhancement in small-holder situations.

Generally, farming system models now in use have some capabilities needed to analyze this Use Case. However, most farming system models are not developed to be easily implemented by non-expert users nor for farms with characteristics different from those for which they were developed. An exception to this is the TOA-MD farming system model (Antle et al., 2014), although that model also needs reliable data from farm surveys to simulate a population of farms in contrast to a particular farm.

##### 3.1.1.2 Input data

It is impractical for Sizani to collect information on a particular farm, go back to her office and work with an analyst to evaluate options for the farmer. Instead, data are needed to describe a range of farming systems so that she could select the combination of biophysical, farming system, and household characteristics from available data. This would include information to allow her to tailor inputs to most closely match the conditions of specific farms. This includes climate, soil, management practice, labor and other inputs available for production and marketing of outputs, typical pest and disease pressures, availability and prices for farming inputs, and other farm, economic, and environmental information.

Generally, sufficient data on the biophysical, environmental, and socio-economic conditions of each farm or for a range of farm typologies in the regions are not available. Although some data, such as climate and soil data, are available, generally these are not organized nor are they sufficiently site-specific that agricultural systems models can readily access them for analysis of specific farms. Although research has shown that some analyses needed to advise a farmer can be made, the availability of input data for agricultural systems models remains a major limitation.

##### 3.1.1.3 Decision support tools

Most existing DSS tools that are available in Apps are focused on relatively narrow issues (e.g., see www.agroclimate.org), such as when to apply a fungicide to a particular crop, when to apply the next irrigation, or how much N fertilizer to apply to a particular crop that will be grown on a particular type of soil in a specific setting. There are few DSS tools that make use of more integrated models to help advisors advise farmers in making farming system decisions (but see Keating et al., 1991, Keating and McCown, 2001). We envision a DSS platform that will connect various models, databases, analysis, and information synthesis tools in an easy-to-use interface for Sizani to set up the analyses and outputs to answer questions about the management of that particular farms' biophysical and socioeconomic situation and the uncertainties in those estimates. Such DSS platforms are possible, but not yet constructed.

### 3.2 Developing and evaluating improved crop and livestock systems for sustainable intensification

Deborah, a plant breeder who is developing drought- and heat-tolerant maize hybrids, would like to evaluate the potential adoption and impacts of new hybrids across widely varying conditions in Africa. She would like to evaluate the potential of new hybrids in complex mixed crop-livestock farming systems relative to sustainable intensification goals (improving productivity, taking into account long term impacts on soils, water and greenhouse gases).

#### 3.2.1 Capabilities and limitations

Models of maize and other crops, livestock, and the farm household are also needed for this Use Case. These models are available for at least partially performing this type of analysis. Starting in the 1980s, several groups began using crop simulation models to evaluate alternative management systems in developing countries (Keating et al., 2003a, Keating et al., 2003b, Uehara and Tsuji, 1998, Penning de Vries et al., 1991). Models used in those efforts were generally based on CERES and other crop models now in DSSAT and on the ORYZA rice model developed by IRRI. More recently, the Global Futures and Harvest Choice CGIAR research projects led by the International Food Policy Research Institute (IFPRI) have used crop and economic models to evaluate the potential benefits of developing new technologies, including new crop varieties (e.g., see Singh et al., 2012, Singh et al., 2013, Singh et al., 2014, Rosegrant et al., 2014). For example, Singh et al. (2012) used the DSSAT CROPGRO groundnut model with climate and soil inputs at six locations in India to evaluate different crop traits being targeted by CGIAR plant breeders. They found that the effect of combining various traits was beneficial, with estimated yield gains varying, depending on location and climate change conditions. Rapid advances in biotechnology and molecular plant breeding are helping researchers incorporate molecular markers and genes into models so that ultimately genetic composition of crops can be used to predict performance of future varieties to help target expensive and time consuming plant breeding efforts (e.g., White and Hoogenboom, 1996, Hoogenboom and White, 2003, Hammer et al., 2006, Messina et al., 2006). The paper by Hwang et al. (this issue) presents some concepts now being explored for next generation crop models.

Similarly, considerable work has been done on farming system models to evaluate options for improving the livelihoods of farmers. These include farm simulation models (e.g., Baudron et al., 2014), optimization models that attempt to select the best combination of enterprises and their management to achieve one or multiple goals of the farmer (usually, maximizing profit, for example, or maximizing utility taking into account attitudes toward risk; e.g., see Nicholson et al., 1994, Herrero et al., 1999, Castelan-Ortega et al., 2003, Waithaka et al., 2006, Gonzalez-Estrada et al., 2008). Also, the Tradeoff Analysis (TOA) model (Antle, 2011, Antle et al., 2014) is currently being used as the basis for model-based impact assessments (Rosenzweig et al., 2013, AgMIP, 2014, Claessens et al., 2012). Furthermore, this approach can incorporate results from crop and livestock models, as well as environmental and social outcome models, and it can be adapted for smallholder or large commercial farming systems.

However, there are important limitations in the capabilities of these models, similar to those mentions in Use Case 1 (e.g., yield limiting soil nutrients, soil physical constraints, and various pest, disease, weed, and other yield reducing factors in farmers' fields not included in current models) Thus, there may be large yield gaps between actual yields in farmers' fields and the potential productivity in those fields (e.g. Van Ittersum et al., 2013, Gustafson et al., 2014). When water, nitrogen, and climate are the major limitations in crop productivity, current models are highly useful, assuming that soil, weather, cultivar, and management input data are available for the analyses. In this Use Case, it is likely that other factors, including other soil nutrients, pests, diseases, and weeds, need to be taken into account. The challenge for next generation models includes not only modeling those factors but also collecting data that describe the production situation with all of the important yield-limiting and reducing factors.

Another question is whether existing biophysical models can predict performance of the wide range of intensification options that may be used by farmers for this Use Case. Some practices would include increased fertilizer, use of disease-resistant varieties, agro-chemicals, modified tillage, increased plant population, soil additives, changing row geometries, and more precise timing and placement of fertilizers. They also could include water harvesting methods (in the field or on the farm), use of drip irrigation, and the use of mulches to reduce soil evaporation and erosion. Although some of these intensification options are widely used, most models do not include this wide range of potential management options. Furthermore, most biophysical models lack components that compute metrics needed to assess sustainable intensification.

Livestock models also have various limitations that need to be addressed in NextGen tools. While our understanding of animal feed requirements is relatively robust, there are still large errors associated with the prediction of feed intake in ruminants, especially under degraded pasture conditions and negligible supplementation. Additionally, few of the models have the capabilities of predicting methane production, and this is becoming more important as assessments of environmental impacts of livestock gain prominence. Gathering more experimental information is needed for validation purposes, especially in the tropics. Another area that merits more work is understanding how climate change is likely to affect livestock systems. We still cannot model the animal and herd responses to increased temperatures, climate variability, and more severe feed fluctuations. Thus, we have limited ability in designing adaptation strategies in livestock systems. There are also few assessments of how feed quality and intake are likely to evolve as a result of climate change. The decision-making process of livestock farmers needs to be better incorporated in whole farm models, with better rules for governing behavioral changes as systems intensify.

Livestock system models need to integrate both biophysical fluctuations in productivity and likely economic responses to these fluctuations in order to get an accurate measure of the impact on the well-being of families who rely on livestock for a substantial part of their income. The demand for livestock products is relatively inelastic, which works in both directions in that a given reduction in the price of meat does not lead to the same proportional increase in demand. Similarly, if there is a substantial increase in the quantity of meat offered for sale, prices will fall by a larger proportion than the quantities offered. The same phenomenon tends to occur in the demand and supply for inputs for livestock production, in particular those deriving from rangeland. The net effect of these price responses to quantity changes is to magnify effect of variations in the supply of livestock products, which can further penalize herdsmen in terms of loss of income and access to money to rebuild herds after drought or other drivers led to loss of herd size.

There are also limitations in socioeconomic models used for evaluating benefits and tradeoffs among different technologies and management of crops and livestock and in managing the farm and its resources. For example, most available economic models simulate average responses and use simple economic-behavioral assumptions. In this Use Case, uncertainty and economic risks must be taken into account. Many economic models can incorporate risk behavior, but doing so adds substantial complexity and imposes high data requirements.

##### 3.2.1.1 Data limitations

Although some limitations are due to models themselves, a basic limitation is in the lack of data for developing, evaluating, and applying models for this Use Case. Although there are useful aggregate agricultural system data (e.g., http://www.nass.usda.gov/) in some countries, those data alone cannot be used to address Deborah's question about sustainable intensification. Data collected by researchers who are studying options for sustainably intensified production systems are sometimes used to test and improve models for addressing intensification approaches.

Limitations discussed in Use Case 1 (Section 3.1) are also relevant here. In addition, there are various types of input data needs for livestock that are difficult to obtain, including species composition in rangelands, diet selection by animals, better spatial representation of feeding practices, adequate parameterization of feed quality parameters and how they change in space and time, improved production systems descriptions, and others.

To address Deborah's needs, input data are needed to characterize intensification technologies for use in the biophysical models as well as to characterize the fields, farm, landscape, hydrology, and ecological components. Some of these limitations are beginning to be addressed, in some countries, through systematic data collection efforts led by the World Bank in its program on designing and implementing Living Standards Measurement Surveys and making those data publicly available.

##### 3.2.1.2 Decision support (DSS) tools

Some progress has been made on information systems that allow one to compute sustainability metrics for specific farming practices. Much is being done by the private sector, and more public-private collaboration in defining and developing improved metrics of sustainability should be considered. However, little has been done to date to produce the types of decision information systems needed to help Deborah advise farmers on sustainable intensification options tailored for specific regions and farming systems. Additional information on limitations in DSS and related knowledge tools is given by Janssen et al. (this issue).

### 3.3 Investment in agricultural development to support sustainable intensification

Stanley is an investment manager for a prominent Foundation, and he needs to evaluate a project for small farms in Kenya that will increase the intensity of production by increasing fertilizer use per hectare on cash crops while maintaining the current sustainable nutrient balance among pasture grasses, crop residues and animal manure.

Economists have developed a benefit-cost analysis approach to evaluate research investments, in principle taking sustainability considerations into account. In this framework, private outcomes (e.g., farm income generated by producing and selling crops and livestock) are combined with the value of “non-market” outcomes, such as maintaining nutrient balances and avoiding environmental contamination, to determine the management strategy that yields the best outcome for farmers and society. In principle, if all policy options could be evaluated in this way, the best option could be identified. To implement this benefit-cost framework however, both quantities and values of marketed goods are needed (e.g., quantity and price of corn produced), as well as quantities and values of non-market outputs (e.g., nutrient concentration in surface water and the environmental or health damages caused by it).

While it is straightforward to measure and value market outcomes, such as the amount and value of maize produced, it is difficult to quantify and value non-market outcomes such as changes in ecosystem services (e.g. water quality or greenhouse gas emissions). With adequate scientific understanding, spatially-relevant data and suitable measurement technologies, it is possible to objectively quantify the non-market outputs (e.g., to track and measure the nutrient concentrations and loadings in water). But in many cases valuing non-market outputs is exceedingly difficult. For example, contamination of water by nutrients such as nitrates may have adverse impacts on human health it may be possible to estimate the magnitude of these effects, but it is difficult to attach a monetary value to health effects that is accepted by the affected people and society. Similarly, ecosystem services such as biodiversity are difficult to quantify and value in monetary terms. For these reasons, strict application of the “benefit-cost analysis” approach to the design of science-based policies faces serious challenges.

An alternative to benefit-cost analysis is “policy tradeoff analysis-TOA” (Crissman et al., 1998, Antle et al., 2014). Rather than attaching monetary values to ecosystem services, the TOA approach defines quantifiable economic, environmental and social “indicators” to assess the well-being of farm households as well as the broader environmental or social impacts of agricultural systems. Alternative investments or policies are evaluated in terms of the interactions among these indicators. In this approach, there is no one “solution” or best policy because different stakeholders may value tradeoffs between outcomes (indicators) differently. However, the TOA approach provides information for stakeholders to make these value judgments.

#### 3.3.1 Input data

Statistically representative data are needed for both bio-physical and economic processes. Special purpose surveys can provide this kind of data, but are costly and time-consuming to generate for each investment decision that Stanley needs to make. As noted in the previous section, a system that combined farm-level decision support with data collection and aggregation would provide a potentially low-cost solution to this problem, and improve both farm-level decision making and the capability for broader investment and policy decision making as well (also see the discussion of these ideas for knowledge product developments in Antle et al., 2016a, Antle et al., 2016b). The feedback loop between the data sensing system and the crop and livestock models based on primary experimental data is an important and untested component for assessing the inevitable gap between experimental results and field implementation. In addition to the collection of farm management data from farm-level decision tools, remote sensing systems from satellites and/or drones could provide data for initializing and updating analysis of a project as it proceeds.

#### 3.3.2 Decision Support Tools

Tools suitable for research investment decision making and policy tradeoff analysis already are in use in some aspects of agricultural research planning and policy design, primarily in industrialized countries such as the United States and the European Union (see Antle et al., 2015a, Antle et al., 2015b for review of examples). Many types of indicators have been developed for policy analysis (Bates and Scarlett, 2013). Various measures of farm household well-being are used, such as farm income and its distribution among geographic regions and among different types of farms. Measures of environmental outcomes and ecosystem services are available from direct measurements and from models, including soil quality and productivity, air and water quantity and quality, greenhouse gas emissions, and wildlife habitat. For example, the US Department of Agriculture has constructed an “environmental benefits index” to assist in the design and implementation of the Conservation Reserve Program (CRP) and the Conservation Security Program (CSP) that combines a number of different environmental indicators into a summary measure.

For analysis in a developing country context, the availability of analytical tools for tradeoff analysis is growing but still substantially limited by lack of adequate data and by the diversity and complexity of small-scale agricultural systems in many parts of the worlds. The integration of model components to address this type of decision problem is similar to those discussed in Section 5.2, but the analysis needs to be extended from the farm to the landscape or regional scale. This type of regional integrated modeling has been implemented in a number of studies, but in each case, a model was developed with data specific to that case (e.g., Stoorvogel et al., 2004, Antle 2008, Antle et al., 2014). These regional integrated assessment models pose a number of additional challenges beyond those described above.

Two approaches have been developed by Antle et al. (2014) for this type of decision support. One is the “Tradeoff Analysis Model” which was designed to function as a stand-alone tool that could integrate specific crop simulation models, environmental process models, and econometric-process simulation models (Stoorvogel et al., 2004). However, the hurdles of data requirements and difficulties of tailoring this type of complex system integration tool to each application proved to be too high for its widespread use. A simpler, generic economic impact assessment approach was subsequently developed that is called the Tradeoff Analysis Model for Multi-Dimensional Impact Assessment, or TOA-MD. This model is a generic framework for integrating data from a number of other models, along with economic data, to model the economic, environmental and social impacts of technology adoption. It is also being used by the AgMIP teams in Africa and South Asia to assess climate impacts (see chapters in Rosenzweig and Hillel, 2014). The AgMIP regional integrated assessment approach (see Section 2.6) utilizes this model in combination with data from climate, crop and livestock models to provide analysis of climate impacts and adaptations. These analyses are then communicated to decision makers through various knowledge products, such as computer-based data visualization tools. Whether this type of regional integrated assessment modeling could be linked to mobile devices and other Apps remains a topic of current research (also see Antle et al., 2016b).

### 3.4 Management support for precision agriculture in the US for profitability, soil conservation, and water quality protection

Greg is a farmer in the US, with a large corn/soybean-based operation and a high level of mechanization fully equipped with auto-tracking system and high-resolution differential GPS. He wants to manage his fields using precision management of input resources to increase efficiency and profits and to reduce environmental risks.

#### 3.4.1 Capabilities and limitations

Strategies to overcome spatial resolution in point-based crop models were first addressed by Basso et al. (2001) and Batchelor et al. (2002) and more recently in Albarenque et al. (2016) and Basso et al. (2016). Such strategies include running point-based models at small scales within a field; geospatial technologies (remote sensing, electrical resistivity tomography, yield mapping) to target the application of models to areas with similar plant responses; and linking point-based models to three-dimensional water flow models to better represent water transport across the landscape (Basso et al., 2001, Basso et al., 2011).

The application of point-based models on small homogenous areas within a field has had limited success in the past due to difficulties in obtaining critical fine scale soil and management information (soil physical and chemical characteristics, including rooting depth, plant population and effective tile drain spacing) necessary to run the models. A current limitation in most crop models is the assumption of uniform plant distribution. Although multiple subfield areas could be simulated, this would require measurements of spatial variations in plant populations, which is usually not available. Visual observations as well as measurements commonly show that plants are not uniformly distributed within fields, and therefore assuming uniformity is unrealistic and a significant source of uncertainty in yield simulations (Ritchie and Basso, 2008, Basso and Ritchie, 2012).

Recent advances on the resolution and availability of remote sensing imagery (satellite, airborne, and Unmanned Aerial Vehicles – UAVs) coupled with a decrease in their associated costs, allow for the collection of timely information on soil and crop variability by examining spatial and temporal patterns of vegetation indices (Ehmke, 2013). Such information can be used to derive inputs for crop models in conjunction with yield mapping analysis to identify areas in the field that are stable over space and time. Crop models can be executed on those areas to provide insights on the reason of variability as well as estimates of potential economic return of variable-rate input prescriptions. Thus, the limitation of availability of spatially-variable data is being overcome through new sensors, communication technologies, and algorithms for producing spatial inputs for use in precision farming as well as statistical and model-based analyses. This is in stark contrast to limitations associated with Use Cases in data-poor regions as noted in Section 3.2.

The assessment of spatial soil water availability is crucial for understanding the interaction of water stress and crop yield variability in agricultural fields, especially now with increased climate variability and extended drought periods. Spatial variation in soil water is often the cause of crop yield spatial variability due to its influence on the uniformity of the plant stand at emergence and in-season water stress. Soil water content is highly variable within a field due to spatial variation in rainfall, topography, soil properties, and vegetation. The ability to simulate spatial soil water content over time is important for models used for agricultural and hydrological systems assessment (i.e. nitrate and pesticide leaching to groundwater, erosion modeling, water logging, and Precision Agriculture applications).

Process-based crop models have proven to be effective in simulating the water balance of soils when the drainage is assumed to be vertical. However, this assumption is incorrect in many fields. For instance, runoff computed by one-dimensional models is not distributed over space, and thus results in inaccurate predictions of surface soil water balance in neighboring areas within a field. The automation of terrain analysis and the use of Digital Terrain Models (DTMs) have made it possible to quantify the topographic attributes of the landscape and to use topography as one of the major driving variables for many hydrological models (Western et al., 1999, Wilson and Gallant, 2000). Basso et al. (2001) developed a spatial soil water balance model that simulates three-dimensional surface and subsurface water flow. The model requires a digital elevation model for partitioning the landscape into a series of interconnected irregular elements, daily weather data, and spatial soil information for the soil water balance simulation. These aspects are considered a serious limitation in crop models and despite their importance have hitherto received limited attention, thus warranting additional improvements and testing.

An example that combines strategic and tactical application of a crop model in a spatial context is described by Basso et al. (2011). A dual-criteria optimization through a tested model could determine the nitrogen rate that minimizes nitrate leaching and increases net revenues for the farmer for three zones within the same field characterized by different yield potentials.

### 3.5 Supplying food products that meet corporate sustainability goals

Jennifer, an economic analyst in a corporate sustainability group, embraces sustainability as the core of their mission: marketing food while conserving resources. She needs to help the corporation's contract farmers with decisions regarding when to plant, when to irrigate and when and how much fertilizer to apply to conserve energy, save water, minimize waste and reduce greenhouse gas emissions in efforts to make these products more sustainable from farm to fork.

An example of the application of crop models to illustrate how reduced N fertilizer rates result in reductions of greenhouse gas emissions (expressed in CO_2_ equivalents) at the field scale are described in Basso et al. (2013) who used the IPCC emission factor approach to model N2O emissions. Shcherbak et al. (2014) showed that IPCC emission factor approach, despite its simplistic approach, is able to closely reproduced measured data of N_2_O emissions. Also, two important aspects to consider in simulating the crop nitrogen uptake and soil nitrogen balance is the initialization of the soil carbon pool (Basso et al., 2011), and to run models in a continuous mode without annually reinitializing soil conditions like soil water and nitrogen content (Basso et al., 2015) in order to properly simulate soil carbon and nitrogen dynamics and their impacts on production and environmental outcomes. Models have also been used to evaluate the energy efficiency in agronomic management (i.e. tillage practices) as reported in Bertocco et al. (2008).

The next generation of crop models with capability of using real time weather and historical climate conditions will be able to identify strategies to optimize the amount of fertilizer used at a particular location and time, soil and weather conditions with the goal of increasing yield and reducing greenhouse gas emissions. Crop models can evaluate the effects of unknown weather conditions and help decide the optimal nitrogen to apply to crops using different amounts within the field using precision agriculture prescription maps. Communication companies have partnered with different high-tech companies to deliver solutions for the meteorological, geo-spatial and operational challenges facing the agriculture industry. Remote monitoring solutions, as an integral part of the Next-Gen model platform, along with advanced cloud services, will help farmers with decisions regarding when to plant, when to irrigate and when and how much N fertilizer to apply.

Some of the large corporate supply chain companies have recently set a goal to improve fertilizer-application efficiency of U.S. row crop farmers in its food supply chain by 30% by 2020. System models can further help these companies by setting emission reduction protocols, benchmarks and baselines to compare emissions among different management strategies, and by incorporating sustainable agricultural criteria into their future plans validating mechanism, including certification to verify that the farmers are meeting sustainability criteria.

There is a need to provide information on the total greenhouse gas, water and other footprints of food systems that are being considered to improve sustainability of future supply from field to fork (Roy et al., 2009). There are existing life cycle analysis (LCA) models that are being used for this purpose, but there are various challenges in providing robust LCA results for complex food systems (Notarnicola et al., 2016). Integration of LCA with biophysical and economic models would provide enable more comprehensive food system sustainability analyses. We are not aware of integrated models or knowledge systems that combine the power of agricultural system models with LCA analyses that would provide strategic foresight indicators of sustainability for use by the food corporation that Jennifer represents, although there have been studies that demonstrate this approach (e.g., Basso et al., 2013).

#### 3.5.1 Input data and decision support tools

Various farm-level data and decision tools are in use, and are evolving rapidly along with innovations in computer power, software, mobile information technologies and technologies for site-specific management (Antle et al., 2015a). A key feature of these tools is that they use both public (e.g., prices, weather forecast and policy information) and private (site- and farm-specific input use, farm size, machinery) data to generate detailed information and outcome-based data that are useful for farm-level management decisions. This information and data can be used to monitor the economic and environmental performance of a farm operation over time and space. The value of these data for improved farm management performance should motivate producers to collect accurate information. In addition, producers increasingly need detailed management data for purposes of quality certification, e.g., for organic or sustainable certification, or to meet regulatory standards.

Various issues need to be addressed to advance the use of these tools for management, certification and related purposes. One issue is how to make data acquisition and analytical tools appropriate for and easy to use by farm-level decision makers (both farmers and organizations that provide management services). Another is how to facilitate the use of data and management tools through effective outreach programs that communicate the value of the tools and the importance of the data for private and public uses. The confidentiality, security and appropriate use of private data when it is shared is another critical issue. Privacy concerns have been the subject of recent discussions among farmers and commodity organizations as they explore the use of new technologies and big-data analytics.

## 4 Discussion

This review of agricultural systems modeling shows that major contributions have been made by various disciplines, addressing different production systems from field to farms, landscapes, and beyond. There are good examples of component models from different disciplines being combined to produce more comprehensive system models that consider biophysical, socioeconomic, and environmental situations to produce a wide range of system responses. For example, crop, livestock, and economic models have been combined to study farming systems as well as to analyze national and global impacts of climate change, policies, or alternative technologies for different purposes. There is a wide range of approaches used in agricultural systems modeling and in the application of those models to scientific and policy questions. Approaches vary according to objectives of developers, their intended uses and data availability.

Developers of agricultural systems models have made good use of theories and concepts from a wide range of disciplines, including agricultural and environmental sciences, ecology, engineering, physics, economics, and statistics. The development of agricultural system models continues to evolve through efforts of many organizations worldwide. Researchers are increasingly interested in contributing to agricultural systems science (e.g., new AgMIP developments, www.agmip.org, and various CGIAR–led programs like the Global Futures project (http://globalfutures.cgiar.org/) and the CCAFS project, https://ccafs.cgiar.org/). Interest in using agricultural system models by the private sector is also increasing.

The Use Cases examined in this special issue demonstrate that a minimum set of component models are needed to develop useful agricultural system models. These component models include, in particular, crop models, livestock models, and farming system models. Crop models combine weather, soil, genetic, and management components to simulate yield, resource use, and outputs of nutrients and chemicals to surrounding water, air, and ecological systems, taking into account weed, pest and disease pressures. They integrate information to predict performance for a range of inputs and practices that apply from subsistence situations to those systems using highly controlled, intensive production technologies and modern varieties.

Similarly, livestock models take into account climate, herd management, feed sources, and breeds. Farming system models integrate various livestock and cropping systems, including their interactions, with economic models that represent the behavior of farm decision makers. These models are needed at the level of the individual operation as well as at the population level so that the bio-physical and socio-economic heterogeneity of systems and their economic, environmental and social impacts can be evaluated by individual farmers as well as policy makers from farm to global scales. Based on this review, we conclude that different platforms for combining models and data for specific purposes are necessary, and that the design of next generation models and data should take into account this need for a range of platforms.

The Use Cases studied included relevant examples across the spectrum of users from small-holder agricultural systems in developing countries to intensive production systems, and from systems supported by agricultural industries to those with little support from the private sector. They include examples that need models and associated data to evaluate technologies at a field or farm scale and others requiring the integration of component models to address socioeconomic, food security, and environmental issues at different scales.

Although the adequacy of available models varied among Use Cases, one limitation was common across all of them, namely the scarcity of data. Data are the foundation for all agricultural systems analyses. This limitation restricts the capabilities of existing models to include factors of importance in most Use Cases, limiting researchers' abilities to evaluate models across wide ranges of conditions (which limits user understanding of and confidence in the reliability of models) and limiting information that can be used as inputs to apply models (Boote et al., 2015, Kersebaum et al., 2014). Data limitations are more important than gaps in conceptual theories and approaches. We argue that limitations of current agricultural system models and tools are more strongly rooted in inadequate data than in knowledge gaps. This limitation restricts users' confidence in model abilities to provide reliable results and thus their use for decisions or policies.

This lack of data is especially severe in less developed regions. This is true for production models of crops and animals as well as economic models across the Use Cases that addressed issues in data-poor areas in Sub Saharan Africa. But it is also clear that many data-rich regions also suffer from lack of accessible and usable data. For example, thousands of agricultural researchers conduct studies each year, comparing technologies and management under specific conditions. Even though those data could be highly useful for developing, evaluating, and using models, they are generally not available except to those involved in specific studies.

There is perhaps one exception to the statement about a common limitation across Use Cases. The capabilities and limitations for management support for precision agriculture (Section 3.4) are different from the others. Although data that characterize spatial variability at a high resolution in individual fields has been limited, this situation is rapidly changing as new sensors and observation platforms (including those on drones and field-mounted platforms) are being developed by the private sector in response to clear business opportunities. This interest by the private sector is likely to rapidly increase the use of agricultural system models to help farmers like Greg (in our example) use precision farming to increase resource use efficiencies, reduce environmental risks, and increase profits. Although the same levels of data collection and precision application of inputs are not likely to be widely used in resource-poor farming situations, advances in technologies are likely to provide additional options in those regions.

Current national and global initiatives are attempting to improve the overall data limitation situation. For example, the Global Open Data for Agriculture and Nutrition (GODAN) initiative is promoting global efforts to make agricultural and nutritionally relevant data available, accessible, and usable for unrestricted use worldwide (http://www.godan.info/). There are over 150 partners in this initiative from national governments, non-governmental, international and private sector organizations who support this effort. It is clear that there is a need for a more focused effort to connect the various agricultural systems modeling, database, data harmonization, open-access, and DSS efforts together, so that the scientific resources being invested in these different initiatives will contribute to compatible set of models, data, and platforms to ensure global public goods. This is critically important, considering that these tools are increasingly needed to ensure that agriculture will meet the food demands of the next 50 to 100 years and will be sustainable environmentally and economically. Efforts are underway to remedy this situation by a number of groups (e.g., Porter et al., 2014). Moreover, as detailed in Antle et al. (this issue), there is a need for strategies such as private-public partnerships to bring together the power of private sector investments with the ongoing research to advance models and modeling tools. This is true for production models of crops and animals as well as economic models across each of the first three Use Cases that address issues in data-poor areas in Sub Saharan Africa.

Finally, based on the current status of models, data, and knowledge systems, a strategy should include the appropriate modification and in some cases re-programming of existing component models that already include many needed capabilities. This would facilitate extension of components that respond to factors that are not currently considered by models, using a range of methods including statistical models, reduced form models, extended databases, and modular models that integrate component submodules. Seavert et al. (2016) suggested that some data limitations could be overcome by integrating farm-level models and knowledge products with landscape-scale data and models. Recent experience in AgMIP demonstrated the value of multiple models indicating that it would not be useful to pursue a goal of producing perfect models for crops, livestock, and farming systems. Although there are excellent prospects for considerable advances in agricultural systems data, models, and knowledge systems, there are inherent limitations in these tools due to irreducible uncertainties in model structures, spatial variability of physical, chemical, genetic, and socioeconomic conditions. These limitations will continue to vary depending on applications, which suggest that future evaluation of capabilities and limitations should be based on well-defined Use Cases. This review indicates that the current state of agricultural systems models is sufficient for some contemporary applications, but that major advances are needed to achieve the next generation of data, models, and knowledge systems to address more complex issues and achieve food security during the next century.

## References

[bb0005] Adams R.M., Rosenzweig C., Peart R.M., Ritchie J.T., McCarl B.M., Glyer J.D., Curry R.B., Jones J.W., Boote K.J., Allen L.H. (1990). Global climate change and U.S. agriculture: an interdisciplinary assessment. Nature.

[bb0010] AFRC (1993). Energy and protein requirements of ruminants. An Advisory Manual Prepared by the AFRC Technical Committee on Response to Nutrients.

[bb0015] AgMIP (2014). Regional Integrated Assessments Handbook. http://www.agmip.org/regional-integrated-assessments-handbook/.

[bb0020] Albarenque S.M., Basso B., Caviglia O.P., Melchiori R.J.M. (2016). Spatio-temporal nitrogen fertilizer response in maize: field study and modeling approach. Agron. J..

[bb0025] Allen V.G., Batello C., Berretta E.J., Hodgson J., Kothmann M., Li X.D., McIvoer J., Milne J., Morris C., Peeters A., Sanderson M. (2011). An international terminology for grazing lands and grazing animals. Grass Forage Sci..

[bb0030] Anselin L. (1988). Spatial econometrics: methods and models. Studies in Operational Regional Science.

[bb0035] Anselin L., Bongiovanni R., Lowenberg-DeBoer J. (2004). A spatial econometric approach to the economics of site-specific nitrogen management in corn production. Am. J. Agric. Econ..

[bb0040] Antle J.M. (1983). Testing the stochastic structure of production: a flexible moment-based approach. J. Bus. Econ. Stat..

[bb0045] Antle J.M. (2011). Parsimonious multi-dimensional impact assessment. Am. J. Agric. Econ..

[bb0050] Antle J.M., Capalbo S.M. (2001). Econometric-process models for integrated assessment of agricultural production systems. Am. J. Agric. Econ..

[bb0055] Antle J.M., Stockle C.O. (2015). Perspectives on climate impacts on crops from agronomic-economic analysis. Review of Environmental Economics and Policy.

[bb2000] Antle J.M., Stoorvogel J.J. (2008). Agricultural carbon sequestration, poverty, and sustainability. Env. & Dev. Econ..

[bb0060] Antle J.M., Stoorvogel J.J., Valdivia R.O. (2014). New parsimonious simulation methods and tools to assess future food and environmental security of farm populations. Philos. Trans. R. Soc. B.

[bb0065] Antle J., Capalbo S., Houston L. (2015). Using Big Data to Evaluate Agro-environmental Policies, Choices 3rd Quarter.

[bb0070] Antle J.M., Valdivia R.O., Boote K.J., Janssen S., Jones J.W., Porter C.H., Rosenzweig C., Ruane A.C., Thorburn P.J., Rosenzweig C., Hillel D. (2015). AgMIP's trans-disciplinary agricultural systems Approach to regional integrated assessment of climate impact, vulnerability and adaptation. Handbook of Climate Change and Agroecosystems: The Agricultural Model Intercomparison and Improvement Project Integrated Crop and Economic Assessments, Part 1.

[bb0075] Antle J.M., Jones J.W., Rosenzweig C. (2016). Towards a new generation of agricultural system models, data, and knowledge products: introduction. Ag. Systems.

[bb0080] Antle J.M., Basso B.O., Conant R.T., Godfray C., Jones J.W., Herrero M., Howitt R.E., Keating B.A., Munoz-Carpena R., Rosenzweig C., Tittonell P., Wheeler T.R. (2016). Towards a new generation of agricultural system models, data, and knowledge products: model design, improvement and implementation. Ag. Systems.

[bb0085] Asseng S., Ewert F., Rosenzweig C., Jones J.W., Hatfield J.L., Ruane A.C., Boote K.J., Thorburn P.J., Rotter R.P., Cammarano D., Brisson N., Basso B., Martre P., Aggarwal P.K., Angulo C., Bertuzzi P., Beirnath C., Challinor A.J., Doltra J., Gayler S., Goldberg R., Grant R., Heng L., Hooker J., Hunt L.A., Ingwersen J., Izaurralde R.C., Kersebaum K.C., Müller C., Naresh Kumar S., Nendel C., O'Leary G., Olesen J.E., Osborne T.M., Palosuo T., Priesack E., Ripoche D., Semenov M.A., Shcherbak I., Steduto P., Stöckle C., Stratonovitch P., Streck T., Supit I., Tao F., Travasso M., Waha K., Wallach D., White J.W., Williams J.R., Wolf J. (2013). Uncertainty in simulating wheat yields under climate change. Nat. Clim. Chang..

[bb0090] Basso B., Ritchie J.T. (2012). Assessing the impact of management strategies on water use efficiency using soil-plant-atmosphere models. Vadose Zone J..

[bb0095] Basso B., Ritchie J.T., Hamilton S.K., Doll J.E., Robertson G.P. (2015). Simulating crop growth and biogeochemical fluxes in response to land management using the SALUS model. The Ecology of Agricultural Landscapes: Long-term Research on the Path to Sustainability.

[bb0100] Basso B., Ritchie J.T., Pierce F.J., Braga R.P., Jones J.W. (2001). Spatial validation of crop models for precision agriculture. Agric. Syst..

[bb0105] Basso B., Gargiulo O., Paustian K., Robertson P.G., Porter C., Grace P.R., Jones J.W. (2011). Procedures for initializing soil organic carbon pools in DSSAT-century model for agricultural systems. Soil Sci. Soc. Am. J..

[bb0110] Basso B., Cammarano D., Fiorentino D., Ritchie J.T. (2013). Wheat yield response to spatially variable nitrogen fertilizer in Mediterranean environment. Eur. J. Agron..

[bb0115] Basso B., Hyndman D.W., Kendall A.D., Grace P.R., Robertson G.P. (2015). Can impacts of climate change and agricultural adaptation strategies be accurately quantified if crop models are annually re-initialized?. PLoS One.

[bb0120] Basso B., Liu L., Ritchie J.T. (2016). A comprehensive review of the CERES-wheat, -maize and-rice models' performances. Adv. Agron..

[bb0125] Bassu S., Brisson N., Durand J.L., Boote K.J., Lizaso J., Jones J.W., Rosenzweig C., Ruane A.C., Adam M., Baron C., Basso B., Biernath C., Boogaard H., Conijn S., Corbeels M., Deryng D., De Sanctis G., Gayler S., Grassini P., Hatfield J., Hoek S., Izaurralde C., Jongschaap R., Kemanian A., Kersebaum K., Müller C., Nendel C., Priesack E., Virginia M., Nin P., Sau F., Shcherbak I., Tao F., Teixeira E., Makowski D., Timlin D., Waha K. (2014). How do various maize crop models vary in their responses to climate change factors?. Glob. Chang. Biol..

[bb0130] Batchelor W.D., Jones J.W., Boote K.J. (1993). Extending the use of crop models to study pest damage. Trans. Am. Soc. Agric. Eng..

[bb0135] Batchelor W.D., Basso B., Paz J.O. (2002). Examples of strategies to analyze spatial and temporal yield variability using crop models. Eur. J. Agron..

[bb0140] Bates, S. and L. Scarlett. 2013. Agricultural Conservation & Environmental Programs: The Challenge of Measuring Performance. Agree Report. Available online: http://www.foodandagpolicy.org/sites/default/files/AGree%20Ag%20Conserv%20and%20Environ-Apr%202013.pdf.

[bb0145] Bates S.L., Zhao J.Z., Roush R.T., Shelton A.M. (2005). Insect resistance management in GM crops: past, present and future. Nat. Biotechnol..

[bb0150] Baudron F., Delmotte S., Corbeels M., Herrera J.M., Tittonell P. (2014). Multi-scale trade-off analysis of cereal residue use for livestock feeding vs. soil mulching in the Mid-Zambezi Valley, Zimbabwe. Agric. Syst..

[bb0155] Berger T., Troost C. (2014). Agent-based modelling of climate adaptation and mitigation options in agriculture. J. Agric. Econ..

[bb0160] Bergez J.E., Raynal H., Launay M., Beaudoin N., Casellas E., Caubel J., Chabrier P., Coucheney E., Dury J., Garcia de Cortazar-Atauri I., Justes E., Mary B., Ripoche D., Ruget F. (2014). Evolution of the STICS crop model to tackle new environmental issues: new formalisms and integration in the modelling and simulation platform. Environ. Model. Softw..

[bb0165] Bertocco M., Basso B., Sartori L., Martin E.C. (2008). Evaluating energy efficiency of site-specific tillage in maize in NE Italy. Bioresour. Technol..

[bb0170] Billari F.C., Fent T., Prskawetz A., Scheffran J. (2006). Agent-based computational modelling: an introduction. Agent-based Computational Modelling.

[bb0175] Bondeau A., Smith P.C., Zaehle S., Schaphoff S., Lucht W., Cramer W., Gerten D., Lotze-Campen H., Muller C., Reichstein M., Smith B. (2007). Modelling the role of agriculture for the 20th century global terrestrial carbon balance. Glob. Chang. Biol..

[bb0180] Booker J.F., Howitt R.E., Michelsen A., Young R. (2012). Economics and the modeling of water resources and policies. Nat. Resour. Model..

[bb0185] Boote K.J., Jones J.W., Mishoe J.W., Berger R.D. (1983). Coupling pests to crop growth simulators to predict yield reductions. Phytopathology.

[bb0190] Boote K.J., Allen L.H., Vara Prasad P.V., Jones J.W., Hillel D., Rosenzweig C. (2010). Testing effects of climate change in crop models. Handbook of Climate Change and Agroecosystems.

[bb0195] Boote K.J., Jones J.W., White J.W., Asseng S., Lizaso J.I. (2013). Putting mechanisms into crop production models. Plant Cell Environ..

[bb0200] Boote K.J., Porter C., Jones J.W., Thorburn P.J., Kersebaum K.C., Hoogenboom G., White J.W., Hatfield J.L., Hatfield J.L., Fleisher D. (2015). Sentinel site data for crop model improvement—definition and characterization. Improving Modeling Tools to Assess Climate Change Effects on Crop Response, Advances in Agricultural Systems Modeling.

[bb0205] Bouman B.A.M., van Keulen H., van Laar H.H., Rabbinge R. (1996). The ‘school of de wit’ crop growth simulation models: a pedigree and historical overview. Agric. Syst..

[bb0210] Brisson N., Gary C., Justes E., Roche R., Mary B., Ripoche D., Zimmer D., Sierra J., Bertuzzi P., Burger P., Bussière F., Cabidoche Y.M., Cellier P., Debaeke P., Gaudillère J.P., Hénault C., Maraux F., Seguin B., Sinoquet H. (2003). An overview of the crop model STICS. Eur. J. Agron..

[bb0215] Brooks-Pollock E., Roberts G.O., Keeling M.J. (2014). A dynamic model of bovine tuberculosis spread and control in Great Britain. Nature.

[bb0220] Carter M.R. (1984). Identification of the inverse relationship between farm size and productivity: an empirical analysis of peasant agricultural production. Oxford Economic Papers New Series.

[bb0225] Castelan-Ortega O., Fawcett R.H., Arriaga C., M., Herrero (2003). A decision support system for smallholder campesino maize-cattle production systems of the Toluca Valley in Central Mexico. 1. Integrating biological and socio-economic models into a holistic system. Agric. Syst..

[bb0230] Challinor A.J., Wheeler T.R., Slingo J.M., Craufurd P.Q., Grimes D.I.F. (2004). Design and optimization of a large-area process-based model for annual crops. Agricultural and Forest Meteorology Journal.

[bb0235] Chambers R.G. (1988). Applied Production Analysis.

[bb0240] Chikowo R., Corbeels M., Tittonell P., Vanlauwe B., Whitbread A., Giller K.E. (2008). Aggregating field-scale knowledge into farm-scale models of African smallholder systems: summary functions to simulate crop production using APSIM. Agric. Syst..

[bb0245] Claessens L., Antle J.M., Stoorvogel J.J., Valdivia R.O., Thornton P.K., Herrero M. (2012). A method for evaluating climate change adaptation strategies for small-scale farmers using survey, experimental and modeled data. Agric. Syst..

[bb0250] Condon L.E., Maxwell R.M. (2013). Implementation of a linear optimization water allocation algorithm into a fully integrated physical hydrology model. Adv. Water Resour..

[bb5000] Coughenour M.B., McKenzie D.H., Hyatt D.E., McDonald V. J (1992). Ecol. Indic.

[bb0255] Crissman C.C., Antle J.M., Capalbo S.M. (1998). Quantifying Tradeoffs in the Environment, Health and Sustainable Agriculture: Pesticide Use in the Andes.

[bb0260] Denisen R.F. (2012). How Understanding Evolution Can Improve Agriculture.

[bb0265] Diaz S., Lavorel S., McIntyre S., Falczuk V., Cadanoves F., Milchunas D.G., Skarpe C., Rusch G., Sternberg M., Noy-Meir I., Landsberg J.J., Zhang W., Clark H., Campbell B.D. (2007). Plant trait responses to grazing – a global synthesis. Glob. Chang. Biol..

[bb0270] Diekman O., Heesterbeek H., Britton T. (2012). Mathematical Tools for Understanding Infectious Disease Dynamics.

[bb0275] D'Odorico P., Okin G.S., Bestelmeyer B.T. (2012). A synthetic review of feedbacks and drivers of shrub encroachment in arid grasslands. Ecohydrology.

[bb0280] Donatelli M., Magarey R.D., Bregaglio S., Willocquet L., Whish J.P.M., Savary S. (2016). Modeling the impacts of pests and diseases on agricultural systems. Agric. Syst..

[bb0285] Dzotsi K.A., Basso B., Jones J.W. (2013). Development, uncertainty and sensitivity analysis of the simple SALUS crop model in DSSAT. Ecol. Model..

[bb0290] Ehmke T. (2013). Unmanned aerial systems for field scouting and spraying. CSA News.

[bb0295] Elliott J., Kelly D., Chryssanthacopoulos J., Glotter M., Jhunjhnuwala K., Best N., Wilde M., Foster I. (2014). The parallel system for integrating impact models and sectors (pSIMS). Environ. Model Softw..

[bb0300] Ewert F., Roetter R.P., Bindi M., Webber H., Trnka M., Kersebaum K.C., Olesen J.E., van Ittersum M.K., Janssen S., Rivington M., Semenov M.A., Wallach D., Porter J.R., Stewart D., Verhagen J., Gaiser T., Palosuo T., Tao F., Nendel C., Roggero P.P., Bartosova L., Asseng S. (2014). Crop modelling for integrated assessment of risk to food production from climate change. Environ. Model. Softw..

[bb0305] FAO (2016). Sustainable Development Goals. http://www.fao.org/sustainable-development-goals/en/.

[bb0310] Flichman G. (2012). Bio-economic Models Applied to Agricultural Systems.

[bb0315] Flichman G., Allen T. (2012). Bio-economic Modeling: State-of-the-art and Key Priorities.

[bb0320] Forrester J.W. (1968). Principles of Systems.

[bb0325] Freer M., Davidson J.L., Armstrong J.S., Donnelly J.R. (1970). Simulation of grazing systems. Proceedings of the XI International Grassland Congress.

[bb0330] Freer M., Moore A.D., Donnelly J.R. (1997). GRAZPLAN: decision support systems for Australian grazing enterprises. I. Overview of the GRAZPLAN project and a description of the MetAccess and LambAlive DSS. Agric. Syst..

[bb0335] Gassman P.W., Sadeghi A.M., Srinivasan R. (2014). Applications of the SWAT model special section: overview and insights. J. Environ. Qual..

[bb0340] Gervois S., de Noblet-Ducoudre N., Viovy N., Ciais P. (2008). Including croplands in a global biosphere model: methodology and evaluation at specific sites. Earth Interactions.

[bb0345] Gonzalez-Estrada E., Rodriguez L.C., Walen V.K., Naab J.B., Koo J., Jones J.W., Herrero M., Thornton P.K. (2008). Carbon sequestration and farm income in West Africa: identifying best management practices for smallholder agricultural systems in northern Ghana. Ecol. Econ..

[bb0350] Gustafson D., Jones J.W., Porter C.H., Hyman G., Edgerton M., Gocken T., Shryock J., Doane M., Ramsey N. (2014). Climate adaptation imperatives: untapped global maize yield opportunities. Int. J. Agric. Sustain..

[bb0355] Hammer G., Cooper M., Tardieu F., Welch S., Walsh B., van Eeuwijk F., Chapman S., Podlich D. (2006). Models for navigating biological complexity in breeding improved crop plants. Trends Plant Sci..

[bb0360] Harou J., Pulido-Velazquez M., Rosenberg D.E., Medellin-Azuara J., Lund J.R., Howitt R.E. (2009). Hydro-economic models: concepts, design, applications, and future prospects. J. Hydrol..

[bb0365] Havlik P., Valin H., Herrero M., Obersteiner M., Schmid E., Rufino M.C., Mosnier A., Thornton P.K., Bottcher H., Conant R.T., Frank S., Fritz S., Fuss S., Kraxner F., Notenbaert A. (2014). Climate change mitigation through livestock system transitions. Proc. Natl. Acad. Sci..

[bb0370] Hazell P.B.R., Norton R.D. (1986). Mathematical Programming for Economic Analysis in Agriculture.

[bb0375] Hazell P.B.R., Scandizzo P.L. (1975). Market intervention policies when production is risky.

[bb0380] Heady E.O. (1957). An econometric investigation of agricultural production functions. Econometrica.

[bb0385] Heady E.O., Dillon J.L. (1964). Agricultural Production Functions.

[bb0390] Herrero M., Fawcett R.H., Dent J.B., Dent J.B. (1996). Integrating simulation models to optimise nutrition and management for dairy farms: a methodology. Livestock Farming Systems: Research, Development, Socio-economics and the Land Manager.

[bb0395] Herrero M., Fawcett R.H., Dent J.B. (1999). Bio-economic evaluation of dairy farm management scenarios using integrated simulation and multiple-criteria models. Agric. Syst..

[bb0400] Herrero M., Gonzalez-Estrada E., Thornton P.K., Quiros C., Waithaka M.M., Ruiz R., Hoogenboom G. (2007). IMPACT: generic household-level databases and diagnostics tools for integrated crop–livestock systems analysis. Agric. Syst..

[bb0405] Herrero M., Havlík P., Valin H., Notenbaert A., Rufino M.C., Thornton P.K., Blümmel M., Weiss F., Grace D., Obersteiner M. (2013). Biomass use, production, feed efficiencies, and greenhouse gas emissions from global livestock systems. Proc. Natl. Acad. Sci..

[bb0410] Holzworth D.P., Huth N.I., deVoil P.G., Zurcher E.J., Herrmann N.I., McLean G., Chenu K., van Oosterom E.J., Snow V., Murphy C., Moore A.D., Brown H., Whish J.P.M., Verrall S., Fainges J., Bell L.W., Peake A.S., Poulton P.L., Hochman Z., Thorburn P.J., Gaydon D.S. (2014). APSIM – evolution towards a new generation of agricultural systems simulation. Environ. Model. Softw..

[bb0415] Holzworth D.P., Snow V., Janssen S., Athanasiadis I.N., Donatelli M., Hoogenboom G., White J.W., Thorburn P. (2015). Agricultural production systems modeling and software: Current status and future prospects. Environ. Model. Softw..

[bb0420] Hoogenboom G., White J.W. (2003). Improving physiological assumptions of simulation models by using gene-based approaches. Agron. J..

[bb0425] Hoogenboom G., Jones J.W., Wilkens P.W., Porter C.H., Boote K.J., Hunt L.A., Singh U., Lizaso J.I., White J.W., Uryasev O., Ogoshi R., Koo J., Shelia V., Tsuji G.Y. (2015). Decision Support System for Agrotechnology Transfer (DSSAT) Version 4.6. http://dssat.net.

[bb0430] Howitt R.E. (1995). Positive mathematical programming. Am. J. Agric. Econ..

[bb0435] Hwang C., Correll M.J., Gezan S.A., Zhang L., Bhakta M., Vallejos C.E., Boote K.J., Clavijo Michelangeli J.A., Jones J.W. (2016). Next generation crop models: a modular approach to model early vegetative and reproductive development of the common bean (*Phaseolus vulgaris* L.). Ag. Systems.

[bb0440] Illius A.W., Allen M.S., Fahey G.C. (1994). Assessing forage quality using integrated models of intake and digestion by ruminants. Forage Quality, Evaluation, and Utilization.

[bb0445] IPCC, Houghton J.T., Jenkins G.J., Ephraums J.J. (1990). Climate change: the IPCC scientific assessment.

[bb0450] Janssen S., Porter C.H., Moore A.D., Athanasiadis I.N., Foster I., Jones J.W., Antle J.M. (2016). Building an open web-based approach to agricultural data, system modeling and decision support. Ag. Systems.

[bb0455] Jenkins M.W., Lund J.R., Howitt R.E., Draper A.J., Msangi S.M., Tanaka S.K., Ritzema R.S., Marques G.F. (2004). Optimization of California's water system: results and insights. J. Water Resour. Plan. Manag..

[bb0460] Johnson, I. 2002. The SGS pasture model: documentation. www.imj.com.au/sgs

[bb8100] Jones J.W., Penning de Vries F.W.T., Teng P., Metselaar K. (1993). Decision support systems for agricultural development. Systems Approaches for Agricultural Development.

[bb0465] Jones J.W., Kenig A., Vallejos C.E. (1999). Reduced state-variable tomato growth model. Trans. ASAE.

[bb0470] Jones J.W., Hoogenboom G., Porter C.H., Boote K.J., Batchelor W.D., Hunt L.A., Wilkens P.W., Singh U., Gijsman A.J., Ritchie J.T. (2003). The DSSAT cropping system model. Eur. J. Agron..

[bb0475] Jones J.W., Antle J.M., Basso B.O., Boote K.J., Conant R.T., Foster I., Godfray H.C.J., Herrero M., Howitt R.E., Janssen S., Keating B.A., Munoz-Carpena R., Porter C.H., Rosenzweig C., Wheeler T.R. (2016). Brief history of agricultural system models. Ag. Systems.

[bb0480] Just R.E., Pope R.D. (1978). Stochastic specification of production function and economic implications. J. Econ..

[bb0485] Just R.E., Zilberman D., Hochman E. (1983). Estimation of multicrop production functions. Am. J. Agric. Econ..

[bb0490] Keating B.A., McCown R.L. (2001). Advances in farming systems analysis and intervention. Agric. Syst..

[bb0495] Keating B.A., Godwin D.C., Watiki J.M., Muchow R.C., Bellamy J.A. (1991). Optimization of nitrogen inputs under climatic risk. Climatic Risk in Crop Production - Models and Management for the Semi-arid Tropics and Sub-tropics.

[bb0500] Keating B.A., Carberry P.S., Hammer G.L., Probert M.E., Robertson M.J., Holzworth D., Huth N.I., Hargreaves J.N.G., Meinke H., Hochman Z., McLean G., Verburg K., Snow V., Dimes J.P., Silburn M., Wang E., Brown S., Bristow K.L., Asseng S., Chapman S., McCown R.L., Freebairn D.M., Smith C.J. (2003). An overview of APSIM, a model designed for farming systems simulation. Eur. J. Agron..

[bb0505] Keating B.A., Gaydon D., Huth N.I., Probert M.E., Verburg K., Smith C.J., Bond W. (2003). Use of modelling to explore the water balance of dryland farming systems in the Murray Darling Basin, Australia. Eur. J. Agron..

[bb0510] Kersebaum K.C., Boote K.J., Jorgenson J.S., Nendel C., Bindi M., Frühauf C., Gaiser T., Hoogenboom G., Kollas C., Olesen J.E., Rotter R.P., Ruget F., Thorburn P.J., Trnka M., Wegehenkel M. (2014). Analysis and classification of data sets for calibration and validation of agro-ecosystem models. Environ. Model. Softw..

[bb0515] Knapp K.C., Olson L.J. (1996). Dynamic resource management: intertemporal substitution and risk aversion. Am. J. Agric. Econ..

[bb0520] Kobayashi M., Howitt R.E., Jarvis L.S., Laca E.A. (2007). Stochastic rangeland use under capital constraints. Am. J. Agric. Econ..

[bb0525] Konandreas P.A., Anderson F.M. (1982). Cattle herd dynamics: an integer and stochastic model for evaluating production alternatives. ILCA Research Report No. 2.

[bb0530] Kucharik C.J. (2003). Evaluation of a process-based agro-ecosystem model (Agro-IBIS) across the U.S. cornbelt: simulations of the inter-annual variability in maize yield. Earth Interact..

[bb0535] Lau L.J., Yotopolous P.A. (1971). A test for relative efficiency and application to Indian agriculture. Am. Econ. Rev..

[bb1000] Lin W., Dean G.W., Moore C.V. (1974). An empirical test of utility vs. profit maximization in agricultural production. Amer. J. Agric. Econ..

[bb0540] Lobell D.B., Schlenker W., Costa-Roberts J. (2011). Climate trends and global crop production since 1980. Science.

[bb0545] Loewer O., Peart M., Curry R.B. (1998). GRAZE: a beef-forage model of selective grazing. Agricultural Systems Modelling and Simulation.

[bb0550] Matson P.A., Parton W.J., Power A.G., Swift M.J. (1997). Agricultural intensification and ecosystem properties. Science.

[bb0555] Maxwell R.M., Putti M., Meyerhoff S.B., Delfs J.O., Ferguson I.M., Ivanov V., Kim J., Kolditz O., Kollet S.J., Kumar M., Lopez S., Niu J., Paniconi C., Park Y.-J., Phanikumar M.S., Shen C., Sudicky E.A., Sulis M. (2014). Surface–subsurface model intercomparison: a first set of benchmark results to diagnose integrated hydrology and feedbacks. Water Resour. Res..

[bb0560] Mendelsohn R., Nordhaus W.D., Shaw D. (1994). The impact of global warming on agriculture: a Ricardian approach. Am. Econ. Rev..

[bb0565] Mérel P., Howitt R.E. (2014). Theory and application of positive mathematical programming in agriculture and the environment. Ann. Rev. Resour. Econ..

[bb0570] Messina C.D., Jones J.W., Boote K.J., Vallejos C.E. (2006). A gene-based model to simulate soybean development and yield responses to environment. Crop Sci..

[bb0575] Milchunas D.G., Lauenroth W.K. (1993). Quantitative effects of grazing on vegetation and soils over a global range of environments. Ecol. Monogr..

[bb0580] Morgan J.A., Milchunas D.G., LeCain D.R., West M.S., Mosier A. (2007). Carbon dioxide enrichment alters plant community structure and accelerates shrub growth in the shortgrass steppe. Proc. Natl. Acad. Sci..

[bb0585] Mundlak Y. (1961). Empirical production function free of management bias. Am. J. Agric. Econ..

[bb0590] Muñoz-Carpena R., Vellidis G., Shirmohammadi A., Wallender W.W. (2006). Evaluation of modeling tools for TMDL development and implementation 392 KB. Trans. Am. Soc. Agric. Eng..

[bb0595] Naab J.B., Singh P., Boote K.J., Jones J.W., Marfo K.O. (2004). Using the CROPGRO-peanut model to quantify yield gaps of peanut in the Guinean savanna zone of Ghana. Agron. J..

[bb0600] Naab J.B., Boote K.J., Jones J.W., Porter C.H. (2015). Adapting and evaluating the CROPGRO-peanut model for response to phosphorus on a sandy-loam soil under semi-arid tropical conditions. Field Crop Res..

[bb0605] Nicholson C.F., Blake R.W., Urbina C.I., Lee D.R., Fox D.G., Van Soest P.J. (1994). Economic comparison of nutritional management strategies for Venezuelan dual-purpose cattle systems. J. Anim. Sci..

[bb0610] Notarnicola B., Sala S., Anton A., McLaren S.J., Saouter E., Sonesson U. (2016). J. Clean. Prod..

[bb0615] NRC (1945). Nutrient Requirements of Dairy Cattle.

[bb0620] NRC (2001). Nutrient Requirements of Dairy Cattle.

[bb6000] Osborne T.M., Lawrence D.M., Challinor A.J., Slingo J.M., Wheeler T.R. (2004). Development and assessment of a coupled crop-climate model. Glob. Chang. Biol..

[bb0625] Osborne T.M., Lawrence D.M., Slingo J.M., Challinor A.J., Wheeler T.R. (2007). Influence of vegetation on local climate and hydrology in the tropics: Sensitivity to soil parameters. Clim. Dyn..

[bb0630] Osborne T.M., Lawrence D.M., Challinor A.J., Slingo J.M., Wheeler T.R. (2007). Development and assessment of a coupled crop–climate model. Glob. Chang. Biol..

[bb0635] Osborne T.M., Slingo J.M., Lawrence D.M., Wheeler T.R. (2009). Examining the interaction of growing crops with local climate using a coupled crop-climate model. J. Clim..

[bb0640] Otter S., Ritchie J.T., Day W., Atkin R.K. (1985). Validation of the CERES-Wheat model in diverse environments. Wheat Growth and Modelling.

[bb9500] Parton W.J. (1993). Observations and modeling of biomass and soil organic matter dynamics for the grassland biome worldwide. Glob. Biogeochem. Cycles.

[bb0645] Penning de Vries F.W.T., van Laar H.H., Kropff M.J. (1991). Simulation and systems analysis for rice production (SARP).

[bb0650] Pinnschmidt H.O., Batchelor W.D., Teng P.S. (1995). Simulation of multiple species pest damage in rice using CERES-rice. Agric. Syst..

[bb0655] Porter C.H., Jones J.W., Adiku S., Gijsman A.J., Gargiulo O., Naab J.B. (2010). Modeling organic carbon and carbon-mediated soil processes in DSSAT v4.5. International Journal of Operational Research.

[bb0660] Porter C.H., Villalobos C., Holzworth D., Nelson R., White J.W., Athanasiadis I.N., Janssen S., Ripoche D., Cufi J., Raes D., Zhang M., Knapen R., Sahajpal R., Boote K., Jones J.W. (2014). Harmonization and translation of crop modeling data to ensure interoperability. Environ. Model. Softw..

[bb0665] Ritchie J.T. (1986). Using computerized crop models for management decisions. Proc. International DLG-Congress for Computer Technology. May 1986. Hannover, Fed. Rep. of Germany.

[bb0670] Ritchie J.T., Basso B. (2008). Water use efficiency is not constant when crop water supply is adequate or fixed: the role of agronomic management. Eur. J. Agron..

[bb0675] Ritchie J.T., Alocilja E.C., Singh U., Uehara G. (1986). IBSNAT and the CERES-Rice model. Proceedings of the Workshop on Impact of Weather Parameters on Growth and Yield of Rice.

[bb0680] Rivington M., Koo J. (2010). Report on the Meta—Analysis of Crop Modelling for Climate Change and Food Security Survey. https://cgspace.cgiar.org/rest/bitstreams/9114/retrieve.

[bb0685] Rosegrant M.W., Koo J., Cenacchi N., Ringler C., Robertson R., Fisher M., Cox C., Garrett K., Perez N.D., Sabbagh P. (2014). Food Security in a World of Natural Resource Scarcity: The Role of Agricultural Technologies.

[bb0690] Rosenzweig C., Hillel D. (2014). Handbook of Climate Change and Agroecosystems: The Agricultural Model Intercomparison and Improvement Project Integrated Crop and Economic Assessments, Part 2.

[bb0695] Rosenzweig C., Parry M.L. (1994). Potential impact of climate change on world food supply. Nature.

[bb0700] Rosenzweig C., Jones J.W., Hatfield J.L., Ruane A.C., Boote K.J., Thorburn P., Antle J.M., Nelson G.C., Porter C., Janssen S., Asseng S., Basso B., Ewert F., Wallach D., Baigorria G., Winter J.M. (2013). The agricultural model intercomparison and improvement project (AgMIP): protocols and pilot studies. Agricultural and Forest Meteorology Journal.

[bb0705] Roy P., Nei D., Orikasa T., Xu Q., Okadome H., Nakamura N., Shiina T. (2009). A review of life cycle assessment (LCA) on some food products. J. Food Eng..

[bb0710] Rufino M., Herrero M., van Wijk M., Hemerink R., de Ridder N., Giller K. (2009). Lifetime productivity of dairy cows in smallholder systems in the highlands of Kenya. J. Anim. Sci..

[bb0715] Savary S., Teng P.S., Willocquet L., Nutter F.W. (2006). Quantification and modeling of crop losses: a review of purposes. Annu. Rev. Phytopathol..

[bb9100] Shcherbak I., Millar N., Robertson G.P. (2014). Global metaanalysis of the nonlinear response of soil nitrous oxide (N2O) emissions to fertilizer nitrogen. PNAS.

[bb0720] Schlenker W., Lobell D.B. (2010). Robust negative impacts of climate change on African agriculture. Environ. Res. Lett..

[bb0725] Schlenker W., Auffhammer M., Hsiang S., Sobel A. (2013). Using weather data and climate model output in economic analyses of climate change. Rev. Environ. Econ. Policy.

[bb0730] Schlesinger W.H., Reynolds J.F., Cunningham G.L., Huenneke L.F., Jarrell W.M., Virginia R.A., Whitford W.G. (1990). Biological feedbacks in global desertification. Science.

[bb0735] Scott N.R., Chen H., Schoen R., Bainbridge W.S., Roco M.C. (2015). Sustainable global food supply. Handbook of Science and Technology Convergence.

[bb0740] Seavert C., Capalbo S., Antle J. (2016). Developing next generation data systems and knowledge products for agricultural producers and policy decision makers. Agric. Syst..

[bb0745] Shrivastava V., Graham W.D., Muñoz-Carpena R., Maxwell R. (2014). Insights on geologic and vegetative controls over hydrologic behavior of a large complex basin - global sensitivity analysis of an integrated parallel hydrologic model. J. Hydrol..

[bb0750] Singh P., Boote K.J., Kumar U., Srinivas K., Nigam S.N., Jones J.W. (2012). Evaluation of genetic traits for improving productivity and adaptation of groundnut to climate change in India. J. Agron. Crop Sci..

[bb0755] Singh P., Nedumaran S., Ntare B.R., Boote K.J., Singh N.P., Srinivas K., Bantilan M.C.S. (2013). Potential benefits of drought and heat tolerance in groundnut for adaptation to climate change in India and West Africa. Mitig. Adapt. Strateg. Glob. Chang..

[bb0760] Singh P., Nedumaran S., Traore P.C.S., Boote K.J., Rattunde H.F.W., Vara Prasad P.V., Singh N.P., Srinivas K., Bantilan M.C.S. (2014). Quantifying potential benefits of drought and heat tolerance in rainy season sorghum for adapting to climate change. Agric. For. Meteorol. J..

[bb0765] Staal S. J., Baltenweck I., Waithaka M.M., DeWolff T., Njoroge L. (2002). Location and uptake: integrated household and GIS analysis of technology adoption and land use, with application to smallholder dairy farms in Kenya. Agric. Econ..

[bb0770] Stehfest E., Heistermann M., Priess J.A., Ojima D.S., Alcamo J. (2007). Simulation of global crop production with the ecosystem model DayCent. Ecol. Model..

[bb0775] Stöckle C.O., Donatelli M., Nelson R. (2003). CropSyst, a cropping systems simulation model. Eur. J. Agron..

[bb0780] Stöckle C.O., Kemanian A.R., Nelson R.L., Adam J.C., Sommer R., Carlson B. (2014). CropSyst model evolution: from field to regional to global scales and from research to decision support systems. Environ. Model. Softw..

[bb0785] Stoorvogel J.J., Antle J.M., Crissman C.C., Bowen W. (2004). The tradeoff analysis model: integrated bio-physical and economic modeling of agricultural production systems. Agric. Syst..

[bb0790] Stringham T.K., Krueger W.C., Shaver P.L. (2003). State and transition modeling: an ecological process approach. J. Range Manag..

[bb3000] Stuth J.W., Schmitt D., Rowan R., Angerer J.P., Zander K. (2003). PHYGROW users guide and technical documentation.

[bb0795] Tack J., Harri A., Coble K. (2012). More than mean effects: modeling the effect of climate on the higher order moments of crop yields. Am. J. Agric. Econ..

[bb0800] Takayama T., Judge G.G. (1964). An interregional activity analysis model of the agricultural sector. Journal of Farm Economics.

[bb0805] Taubert F., Frank K., Huth A. (2012). A review of grassland models in the biofuel context. Ecol. Model..

[bb0810] Taylor. E, and I Adelman. (2006) Village economies. The Design, Estimation and Use of Village wide Economic Models. Cambridge: Cambridge University Press, 2006.

[bb0815] Tedeschi L., Cavalcanti L., Fonseca M., Herrero M., Thornton P.K. (2014). The evolution of dairy cattle models for predicting milk production: an agricultural model intercomparison and improvement project (AgMIP) for livestock. Anim. Prod. Sci..

[bb0820] Thorburn P., Boote K., Hargreaves J., Poulton P., Jones J., Hillel D., Rosenzweig C. (2014). Crop systems modeling in AgMIP: a new protocol-driven approach for regional integrated assessments. Handbook of Climate Change and Agroecosystems: Agricultural Model Intercomparison and Improvement Project Integrated Crop and Economic Assessments (Vol. 3 & 4). ICP Series on Climate Change Impacts, Adaptation, and Mitigation.

[bb5200] Thornley J. (1997). Temperate Grassland Responses to Climate Change: an Analysis using the Hurley Pasture Model. Ann. Bot..

[bb0825] Thornton P.K., Herrero M. (2001). Integrated crop-livestock simulation models for scenario analysis and impact assessment. Agric. Syst..

[bb0830] Tittonell P., Zingore S., M. v.W., Corbeels M.C., Giller K.E. (2007). Exploring diversity in soil fertility management of smallholder farms in western Kenya. II. Within-farm variability in resource allocation, nutrient flows and soil fertility status. Field Crop Res..

[bb0835] Uehara G., Tsuji G.Y., Tsuji G.Y., Hoogenboom G., Thornton P.K. (1998). Overview of IBSNAT. Chapter 1, pp. 1–7. Understanding Options for Agricultural Production.

[bb0840] Van Arendonk J.A.M., Dijkhuizen A.A. (1985). Studies on the replacement policies in dairy cattle. III. Influence of variation in reproduction and production. Livest. Prod. Sci..

[bb0845] Van Auken O.W. (2000). Shrub invasions of North American semiarid grasslands. Annu. Rev. Ecol. Syst..

[bb0850] van Ittersum M.K., Leffelaar P.A., van Keulen H., Kropff M.J., Bastiaans L., Goudriaan J. (2003). On approaches and applications of the Wageningen crop models. Eur. J. Agron..

[bb0855] Van Ittersum M.K., Cassman K.G., Grassini P., Wolf J., Tittonell P., Hochman Z. (2013). Yield gap analysis with local to global relevance—a review. Field Crop Res..

[bb0860] van Wijk M.T., Tittonell P., Rufino M.C., Herrero M., Pacini C., Ridder N., de Giller K.E. (2009). Identifying key entry-points for strategic management of smallholder farming systems in sub-Saharan Africa using the dynamic farm-scale simulation model NUANCES-FARMSIM. Agric. Syst..

[bb0865] van Wijk M., Rufino M., Enahoro D., Parsons D., Silvestri S., Valdivia R.O., R., Herrero M. (2014). Farm household models to analyse food security in a changing climate: A review. Global Food Security.

[bb0870] Vesk P.A., Westoby M. (2001). Predicting plant species' responses to grazing. J. Appl. Ecol..

[bb0875] Waithaka M.M., Thornton P.K., Herrero M., Shepherd K.D. (2006). Bio-economic evaluation of farmers' perceptions of viable farms in western Kenya. Agric. Syst..

[bb0880] Western A.W., Grayson R.B., Bloschl G., Willgoose G.R., McMahon T.A. (1999). Observed spatial organization of soil moisture and its relation to terrain indices. Water Resour. Res..

[bb0885] Whish J.P.M., Herrmann N.I., White N.A., Moore A.D., Kriticos D.J. (2015). Integrating pest population models with biophysical crop models to better represent the farming system. Environ. Model. Softw..

[bb0890] White J.W., Hoogenboom G. (1996). Simulating effects of genes for physiological traits in a process-oriented crop model. Agron. J..

[bb0895] Williams J.R., Jones C.A., Kiniry J.R., Spanel D.A. (1989). The EPIC crop growth model. Trans. Am. Soc. Agric. Eng..

[bb0900] Willocquet L., Elazegui F.A., Castilla N., Fernandez L., Fischer K.S., Peng S., Teng P.S., Srivastava R.K., Singh H.M., Zhu D., Savary S. (2004). Research priorities for rice disease and pest management in tropical Asia: a simulation analysis of yield losses and management efficiencies. Phytopathology.

[bb0905] Wilson J.P., Gallant J.C. (2000). Terrain Analysis: Principles and Applications.

[bb0910] Wolfe M.L., Ting K.C., Scott N., Sharpley A., Jones J.W., Verma L. (2016). Engineering solutions for food-energy-water systems: it is more than engineering. J. Environ. Stud. Sci..

[bb0915] Woodward S.J.R., Peart R.M., Curry R.B. (1998). Dynamical systems models and their application to optimizing grazing management. Agricultural Systems Modeling and Simulation.

